# Inhibition of Aryl Hydrocarbon Receptor (AhR) Expression Disrupts Cell Proliferation and Alters Energy Metabolism and Fatty Acid Synthesis in Colon Cancer Cells

**DOI:** 10.3390/cancers14174245

**Published:** 2022-08-31

**Authors:** Martina Karasová, Jiřina Procházková, Zuzana Tylichová, Radek Fedr, Miroslav Ciganek, Miroslav Machala, Zdeněk Dvořák, Barbora Vyhlídalová, Iveta Zůvalová, Jiří Ehrmann, Jan Bouchal, Zdeněk Andrysík, Jan Vondráček

**Affiliations:** 1 Department of Cytokinetics, Institute of Biophysics of the Czech Academy of Sciences, 61265 Brno, Czech Republic; 2 Department of Experimental Biology, Faculty of Science, Masaryk University, 62500 Brno, Czech Republic; 3 International Clinical Research Center, St. Anne’s University Hospital Brno, 65691 Brno, Czech Republic; 4 Department of Pharmacology and Toxicology, Veterinary Research Institute, 62100 Brno, Czech Republic; 5 Department of Cell Biology and Genetics, Faculty of Science, Palacký University, 78371 Olomouc, Czech Republic; 6 Department of Clinical and Molecular Pathology, Institute of Molecular and Translational Medicine, Faculty of Medicine, Palacký University and University Hospital, 77900 Olomouc, Czech Republic; 7 Linda Crnic Institute for Down Syndrome, School of Medicine, University of Colorado Anschutz Medical Campus, Aurora, CO 80045, USA; 8 Department of Pharmacology, School of Medicine, University of Colorado Anschutz Medical Campus, Aurora, CO 80045, USA

**Keywords:** colon cancer cells, AhR, metabolism, proliferation, fatty acid synthesis, Akt pathway

## Abstract

**Simple Summary:**

Cancer cells undergo metabolic modifications in order to meet their high energetic demand. The aryl hydrocarbon receptor (AhR) is a ligand-activated transcriptional factor primarily known as a xenobiotic sensor. However, this receptor seems to have a wide range of physiological roles in many processes including cell proliferation, migration or control of immune responses. AhR is often overexpressed in tumor cells of various tissue origin, and several studies have indicated that AhR may also contribute to regulation of cellular metabolism, including synthesis of fatty acids (FA), one of the major steps in metabolic transition. Potential links between the AhR and the control of tumor cell proliferation and metabolism thus deserve more attention.

**Abstract:**

The aryl hydrocarbon receptor (AhR) plays a wide range of physiological roles in cellular processes such as proliferation, migration or control of immune responses. Several studies have also indicated that AhR might contribute to the regulation of energy balance or cellular metabolism. We observed that the AhR is upregulated in tumor epithelial cells derived from colon cancer patients. Using wild-type and the corresponding AhR knockout (AhR KO) variants of human colon cancer cell lines HCT116 and HT-29, we analyzed possible role(s) of the AhR in cell proliferation and metabolism, with a focus on regulation of the synthesis of fatty acids (FAs). We observed a decreased proliferation rate in the AhR KO cells, which was accompanied with altered cell cycle progression, as well as a decreased ATP production. We also found reduced mRNA levels of key enzymes of the FA biosynthetic pathway in AhR KO colon cancer cells, in particular of stearoyl-CoA desaturase 1 (*SCD1*). The loss of AhR was also associated with reduced expression and/or activity of components of the PI3K/Akt pathway, which controls lipid metabolism, and other lipogenic transcriptional regulators, such as sterol regulatory element binding transcription factor 1 (*SREBP1*). Together, our data indicate that disruption of AhR activity in colon tumor cells may, likely in a cell-specific manner, limit their proliferation, which could be linked with a suppressive effect on their endogenous FA metabolism. More attention should be paid to potential mechanistic links between overexpressed AhR and colon tumor cell metabolism.

## 1. Introduction

Colorectal cancer (CRC) is the third most commonly diagnosed malignancy, and the second leading cause of cancer death, worldwide [1]. Over 50% of CRC cases can be attributed to modifiable risk factors, such as smoking, unhealthy food, high alcohol consumption, lack of physical activity, or excess body weight [2]. Over the recent years, it became evident that cancer development, including CRC, is associated with a transformation of cellular metabolism enabling cancer cells to utilize alternate energy resources [3,4,5]. The changes in cancer cell metabolism lead also to altered production of various lipids, which serve as energy sources for rapidly proliferating tumor cells [3]. The association between CRC and alterations in lipids observed in colon cancer cells indicate that disruption of lipid metabolism, which is involved both in CRC initiation and its progression, may represent a targetable vulnerability in this disease [6].

The aryl hydrocarbon receptor (AhR) is a ligand-activated transcription factor primarily known as a xenobiotic sensor and transcriptional regulator of genes involved in metabolism of xenobiotics, including cytochrome P450 family 1 enzymes [7]. The AhR ligands include exogenous toxic pollutants, such as polycyclic aromatic hydrocarbons or polychlorinated dibenzo-*p*-dioxins and dibenzofurans. The AhR can also be activated by multiple phytochemicals and endogenous ligands, in particular by various tryptophan metabolites [8,9]. The AhR has been implicated not only in the metabolism of xenobiotics, but in a much wider range of cellular and physiological processes, which include control of cell proliferation, migration, immune system functions and many others [10,11,12,13,14,15,16]. The AhR null (AhR KO) mice exhibit a range of developmental disorders, such as e.g., liver abnormalities or alterations of immune system and hematopoiesis [17,18].

The role of the AhR in human cancer remains less clear, as in some tumor types, the AhR plays a role of a tumor suppressor, while in others, it behaves as an oncogenic factor [19]. In colorectal cancer, the role of AhR has been reported to be ambiguous. Studies with AhR KO mice documented a higher colorectal tumor incidence in the AhR-deficient animals than in their wild-type counterparts [20]. In addition, tumor occurrence in an azoxymethane-induced colorectal carcinogenesis model is higher in AhR KO mice [21], and the loss of AhR in intestinal epithelial cells promotes carcinogenesis in mice via upregulating Wnt signaling [22]. These data seem to indicate that the AhR suppresses colorectal carcinogenesis. However, the impact of the AhR could be cancer stage-dependent, and other evidence suggests that AhR is overexpressed in cancer colon tissue in comparison with healthy tissue [23,24,25]. Natural AhR ligands induce an AhR-dependent apoptosis in cancer cells [26,27]. Other factors, including diet, may also modulate the impact of the AhR on colon carcinogenesis [28]. Finally, the AhR has been also shown to be associated with Src-mediated epidermal growth factor-induced stimulation of colon cancer cell proliferation, as well as with sustainability of indoleamine-2,3-dioxygenase expression and activity, which catalyzes the first step in the kynurenine pathway (that in turn leads to formation of endogenous AhR ligands), which also contributes to tumorigenesis [29,30]. Therefore, potential impact of AhR on colon cancer cell behavior is not fully clear.

The AhR may impact cell proliferation through diverse mechanisms, which have been studied primarily in cancer cells. Its modes of action include the interactions of the AhR with retinoblastoma (Rb) protein [31] or regulation of expression of cyclin-dependent kinase inhibitors, such as p27^Kip1^ [32]. The AhR has been shown to act in synergy with Rb to repress E2F-dependent gene expression by displacing histone acetyltransferase p300 from E2F-dependent promoters, and to slow down cell cycle progression, particularly in the G1- to S-phase transition, thus promoting G1 arrest [33]. AhR activation may also inhibit cell cycle progression via inducing p27 ^Kip1^ expression [32]. The accumulation of p27^Kip1^ leads to its increased association with cyclin E/cyclin-dependent kinase 2 complex, thereby inhibiting phosphorylation of Rb [11,34]. Nevertheless, the AhR may interact with other transcriptional regulators, and thus indirectly contribute to entry of cells into the cell cycle. For instance, the AhR has been shown to induce expression of several growth factors inducing cell proliferation [7,35,36]. The overall impact of the AhR on cell proliferation and cell cycle progression is likely to be tissue specific, and it can be affected by factors involved in cell transformation, as well as by the tumor microenvironment.

Given the extensive cross-talk of the AhR with other oncogenic signaling pathways (including, e.g., PI3K/Akt, c-myc or Wnt/β-catenin-dependent signaling), there are multiple unknown factors, which may determine tumor suppressor or oncogenic role of AhR in a given tissue/cell-specific context [37,38,39]. The current evidence suggests that the AhR may also impact cell metabolism through some of these interactions [40]. Of note, the AhR has been proposed to regulate synthesis of fatty acids (FAs). 2,3,7,8-Tetrachlorodibenzo-*p*-dioxin (TCDD), a potent AhR agonist, induces hepatic steatosis and metabolic dysfunction [41,42]. The TCDD treatment in rodents has been shown to alter regulation of FA synthesis and glucose metabolism genes, including two critical lipogenic genes, fatty acid synthase (FASN) and acetyl-CoA carboxylase (ACC) [43]. TCDD-induced steatosis has been shown to be associated with induction of stearoyl-CoA desaturase 1 (*SCD1*) expression, and corresponding alterations of hepatic FA composition [44]. The constitutively activated AhR mutant form has also been shown to induce upregulation of the CD36 FA transporter and to suppress FA oxidation [45]. The transcript levels of CD36, as well as hepatic lipogenic genes regulated by the transcription factor sterol regulatory element binding protein 1c (SREBP1c), such as FASN and ACC, have been found to be significantly lower in the liver of AhR KO mice [46]. Collectively, multiple lines of evidence indicate that the AhR might impact endogenous cell metabolism, including the metabolism of FAs. 

The above-mentioned studies seem to envision multiple roles of the AhR in control of both cell proliferation and cell metabolism in tumor cells. However, the relationship of the AhR and the control of these important processes in colon cancer cells has been only partly explored. The primary goal of our study was thus to evaluate functional role(s) of the AhR in the control of proliferative and metabolic behavior of colon cancer cells, with a focus on FA metabolism genes, since the AhR seems to be upregulated in parallel with FA synthesis genes in colon tumors [47,48]. 

## 2. Materials and Methods

### 2.1. Cell Culture

Human cancer cell lines derived from colorectal cancer (HCT116 and HT-29) were obtained from American Type Culture Collection (ATCC, Rockville, MD, USA). The generation of AhR KO HT-29 cells has been reported previously [49]. The preparation of AhR KO HCT116 cells is described below. HCT116 PTEN-deficient cells [50] were kindly provided by T. Waldman (Georgetown University School of Medicine, Washington, DC, USA). All cell lines were cultured in McCoy’s 5A medium, supplemented with 1× penicillin/streptomycin mix and 10% inactivated fetal bovine serum (iFBS; Thermo Fisher Scientific, Waltham, MA, USA). Cells were cultured at 37 °C in 5% CO_2_ and 95% humidity, and they were passaged twice per week using trypsin/EDTA (0.05/0.02%) for cell detachment. HepaRG AhR KO and control 5F Clone cells were obtained from Sigma Aldrich (Prague, Czech Republic). The cells were cultured as recommended by the manufacturer.

### 2.2. Generation of AhR Knock-Out HCT116 Cells

To prepare HCT116 AhR KO cells, we used CRISPR/Cas9 technique. Briefly, we screened single-cell knockout clones obtained with gRNA (AAGTCGGTCTCTATGCCGCT) targeting second exon of *AHR* gene (ENST00000242057.9). AhR levels in individual clones were determined by Western blotting, and selected candidate clones were validated by sequencing of the disrupted locus. Control gRNA cell line was generated from transfecting the cells with empty CRISPR/Cas9 vector without gRNA.

### 2.3. Cell Painting and Morphology Analysis

Cells were seeded at a density of 50,000 cells per cm^2^ into 384-well black plate (4 wells per each cell line) and grown for 48 h at standard conditions. Next, the cells were stained with Hoechst 33342, concanavalin A, SYTO14, phalloidin, wheat germ agglutinin and MitoTracker as described previously [51], using epMotion 5075 (Eppendorf, Hamburg, Germany) liquid handling workstation. Images were acquired using ImageXpress Micro (Molecular Devices, San Jose, CA, USA) fluorescence microscope (40× objective). Briefly, 5 fluorescence channels, using DAPI, Cy3, GFP, TxRed and Cy5 filters, were captured from 35 sites in each well, or 1000 cells per well were acquired with adaptive acquisition set-up. Spillovers of fluorescence from different channels were compensated by subtraction of dim signal based on single-stained compensation controls. Representative images were artificially colored, and scale bar was burned into images using ImageJ 1.51p (NIH, Bethesda, MD, USA). Acquired images were processed based on the original cell painting protocol [51]. Objects of nuclei, cytoplasm and whole cell areas were segmented, and 1785 features were calculated from 5 fluorescence channels on these three objects, using CellProfiler v2.2.0 software. Data were scaled, missing data and constant values in specific features were removed before principal component analysis, and t-SNE analyses (perplexity 1605, number of iterations 500, minimum cost value 0.5) were performed. The plots were generated in R (RStudio, Inc., Boston, MA, USA).

### 2.4. Immunohistochemistry

The patient cohort and preparation of tumor samples are described in detail in our previous study [47]. Fresh colon tumor tissues were harvested during elective tumor removal surgery; the healthy autologous tissue removed alongside the tumor was used as non-tumor control. The study was conducted according to the guidelines of the Declaration of Helsinki and was approved by the Ethics Committee of the University Hospital Olomouc. Formalin-fixed paraffin-embedded tissue samples were stained according to standard techniques with the mouse monoclonal antibody specific for AhR (clone RPT1, dilution 1:500, Thermo Fisher Scientific). Antigen retrieval was performed with CC1 (Roche Diagnostics, Mannheim, Germany) at 95 °C for 64 min. Target expression was assessed semi-quantitatively by a pathologist using the histoscore method, where the percentage of positive cells (0–100%) was multiplied by staining intensity (0–3), which resulted in a final score between 0 and 300 (H-score).

### 2.5. Cell Proliferation and Cell Cycle Analyses

Cells were seeded at the density 3000 cells per well on 96-well plate. After 72 h, cell proliferation assay was performed using CyQUANT direct cell proliferation kit (Thermo Fisher Scientific), according to the manufacturer’s instructions. Measurement of fluorescence was performed with MBG Fluostar Galaxy reader (MTX Lab systems, Vienna, VA, USA). Alternatively, cells were trypsinized and counted using CASY TT electronic cell counter (Roche Diagnostics). For detection of cell cycle, cells were seeded at a density of 30,000 cells per cm^2^ on 12-well plates in triplicates. After 72 h, cell cycle progression was determined in propidium iodide-stained cell nuclei by FACSVerse (BD Biosciences, Franklin Lakes, NJ, USA). The cell cycle distribution was analyzed with ModFit LT software (Verity Software House, Topsham, ME, USA).

### 2.6. Dual Luciferase Assay (TOP/FOP)

HCT116 cells were plated at a density of 40,000 per cm^2^ in 24-well plates in a complete growth medium without antibiotics. After 24 h cultivation, cells were transiently transfected with pRL-TK vector encoding Renilla luciferase (for transfection efficiency control), and either Super 8× TOPFlash or Super 8× FOPFlash reporter constructs [52]. Luciferase assays were carried out using the Dual-Luciferase Reporter Assay System (Promega, Madison, WI, USA). The chemiluminescence was measured with luminometer LM-01T (Immunotech, Prague, Czech Republic). The firefly luciferase activity in each well was normalized to the corresponding Renilla luciferase activity readout, in order to control for transfection efficiency.

### 2.7. Real-Time Quantitative RT-PCR (RT-qPCR)

Colon cancer cells were seeded at a density 30,000 cells per cm^2^ in 6-well plates and 48 h after seeding cells were washed twice with cold PBS and lysed with cell lysis buffer. Total RNA was isolated from cells using the NucleoSpin^®^RNA II purification kit (Macherey-Nagel, Düren, Germany). The amplification of the samples was carried out with Superscript III Platinum One-Step qRT-PCR kit (Invitrogen, Carlsbad, CA, USA). Modified conditions were used for HepaRG cells. Here, TRI Reagent^®^ (Sigma Aldrich) was used for total RNA isolation, and two-step RT-qPCR was performed using on the Lightcycler 480 II thermocycler (Roche Diagnostics). The concentration and purity of total RNA was determined spectrophotometrically. All reactions were performed in triplicates, and changes in gene expression were calculated by using the comparative threshold cycle method [53]. Primers and the respective probes were provided by Roche Diagnostics. For primer sequences and UPL probes list, see Supplementary Table S1.

### 2.8. Western Blotting

Cells were washed twice with cold phosphate-buffered saline (PBS) (4 °C), and whole cell lysates were prepared in sodium dodecyl sulphate (SDS) lysis buffer (1% SDS, 10% glycerol, 100 mM Tris pH 7.4, Protease Inhibitor Cocktail (Calbiochem, San Diego, CA, USA), 1 mM Na_3_VO_4_, 1 mM NaF), sonicated and heated for 10 min at 90 °C. Protein concentration was determined with Bio-Rad DC Protein Assay (Bio-Rad Laboratories Inc., Hercules, CA, USA). The isolation and preparation of EpCAM^+^ cells from tumors and normal colon mucosa have been described previously [48]. The protein samples were separated by SDS-PAGE and transferred onto polyvinylidene difluoride membrane (Millipore). Specific proteins were detected with primary antibodies (see Supplementary Materials Table S2 for the full list of antibodies used in the present study). The mouse monoclonal antibody against β-actin (A5441, Sigma-Aldrich) was used as loading control. Anti-rabbit and anti-mouse secondary antibodies, conjugated with horse-radish peroxidase, and ECL-Plus reagent were purchased from GE Healthcare (Little Chalfont, UK), and they were used according to the manufacturer’s instructions.

### 2.9. Detection of ATP Production with SeaHorse Analyzer and Detection of Glucose Levels in Cell Culture Media

The ATP production in wild-type and AhR KO cells was explored using the Seahorse HS XFp extracellular flux analyzer using the XFp real-time ATP rate assay kit (Agilent Technologies, Santa Clara, CA, USA), according to the manufacturer’s instructions. For detection of glucose in cell culture media, cells were seeded in 96-well plate at the density of 1000 cells per well. After 24 h, the medium was exchanged, and wild-type cells were treated with DMSO as control, or with AhR antagonist CH-223191 (10 mM). After 48 h, medium was collected and glucose content was analyzed using glucose colorimetric/fluorometric assay kit (BioVision, Waltham, MA, USA) according to the manufacturer’s instructions. DNA content (CyQUANT assay) was used for normalization.

### 2.10. Analysis of Lipid and FA Content

Total levels of neutral lipids and phospholipids were measured by flow cytometry using LipidTOX staining. Briefly, cells were seeded at density 30,000 cells per cm^2^ in 6-well plates. HCS LipidTOX™ Green Phospholipidosis Detection Reagent (Thermo Fisher Scientific) was added to cells 48 h, and HCS LipidTOX™ Deep Red Neutral Lipid Stain (Thermo Fisher Scientific) was added to cells 1 h prior to their analysis. Cells were detached, washed in PBS and analyzed on FACSVerse. Median Fluorescence Intensity (MFI) values were then estimated.

The analyses of FAs were performed by gas chromatography/mass spectrometry (GC/MS) method described in our previous study [47]. All standards, chemicals and solvents were obtained from Sigma-Aldrich. In brief, cell extracts were evaporated to dryness, and we then performed transesterification of FAs, as previously described [54]. The FA methyl esters were then extracted twice with 1 mL of hexane. Combined extracts were again evaporated to dryness, and they were finally dissolved in 200 μL of 2,2,4 trimethylpentane. We injected 1 μL of sample to the GC/MS. GC/MS analysis was performed using a combination of determination of retention times and relative abundances of the selected ions. GC separation was performed in an Omegawax-fused silica capillary column (30 × 0.25 mm I.D., 0.25 μm, Supelco, Bellefonte, PA, USA), using helium as a carrier gas (at the column head pressure of 70 kPa). For detection and identification of the analytes, we employed an ion trap mass spectrometer Saturn 2100T (Varian, Walnut Creek, USA). The levels of FA were expressed as nmol per 10^6^ cells. For estimation of theoretical SCD enzymatic activity, we then calculated ratios of C16:1n7/C16:0 and C18:1n9/C18:0 FAs [47].

### 2.11. Statistical Analysis

All data presented in the study are plotted as mean ± SD of at least three independent experiments. Statistical analysis was performed with GraphPad Prism 5 (GraphPad Software, San Diego, CA, USA) applying Student’s *t* test for pairwise comparisons (threshold value of *p* < 0.05 or *p* < 0.01).

## 3. Results

### 3.1. AhR Is Overexpressed in CRC Tumor Samples and in Isolated Colon Cancer Epithelial Cells

**Figure 1:**
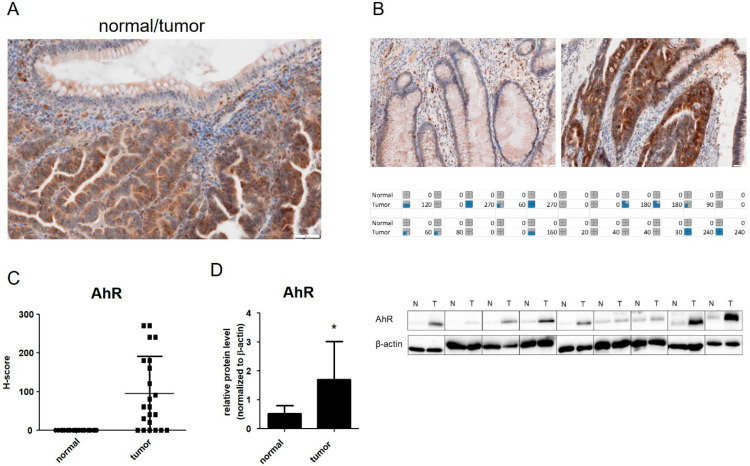
AhR protein levels are increased in human colon cancer tissue. (**A**) A representative image of AhR staining in colon tumor; (**B**) a comparison of AhR staining between normal (left) and tumor (right) colon tissue. H-scores were calculated based on intensity and positivity values. Pictograms at the bottom of the panel document staining of all formalin-fixed paraffin-embedded tissue specimens (n = 22; grey, H-score 0–59; blue, H-score in quartiles up to 119, 179, 239 and 300); (**C**) H-score values comparing AhR staining in normal and tumor tissue. (**D**) AhR protein levels were determined by Western blotting in representative samples of isolated tumor and non-tumor colon epithelial cells (n = 10), using anti-human AhR rabbit monoclonal antibody, with β-actin being used as loading control. Representative Western blotting images are shown at the right. For original blots, see Supplementary File S1. Relative AhR levels determined by densitometry and normalized to -actin are shown at the left. * denotes a significant difference (*p* < 0.05) between AhR protein levels in tumor and non-tumor cells.

Several studies have indicated that the AhR is overexpressed in colon tumor tissue [23,24,25]. Such reports are in agreement with our previous study documenting increased AhR mRNA levels in a cohort of CRC patients [48]. As disparities between transcript levels and protein expression are common in tumor tissue [55], we decided to validate our finding by staining the tumor tissue for AhR protein and comparing the intensity of its staining with the adjacent normal colon mucosa. We observed that the AhR levels were higher in colon cancer tissue, as compared with the adjacent healthy tissue (Figure 1A–C). Importantly, the elevated AhR protein levels were also found in isolated EpCAM^+^ tumor epithelial cells (Figure 1D), which were prepared from a subset of patient samples diagnosed as colon adenocarcinomas, as well as from the corresponding adjacent normal tissue collected during operations [48]. This confirmed that the AhR is upregulated directly in colon cancer epithelial cells, and that the observed upregulation in whole tumor tissue was not linked with stromal cells [23], or with other cell types infiltrating the tumor tissue.

### 3.2. Loss of the AhR Reduces Proliferative Rate of Colon Cancer HCT116 and HT-29 Cells

**Figure 2:**
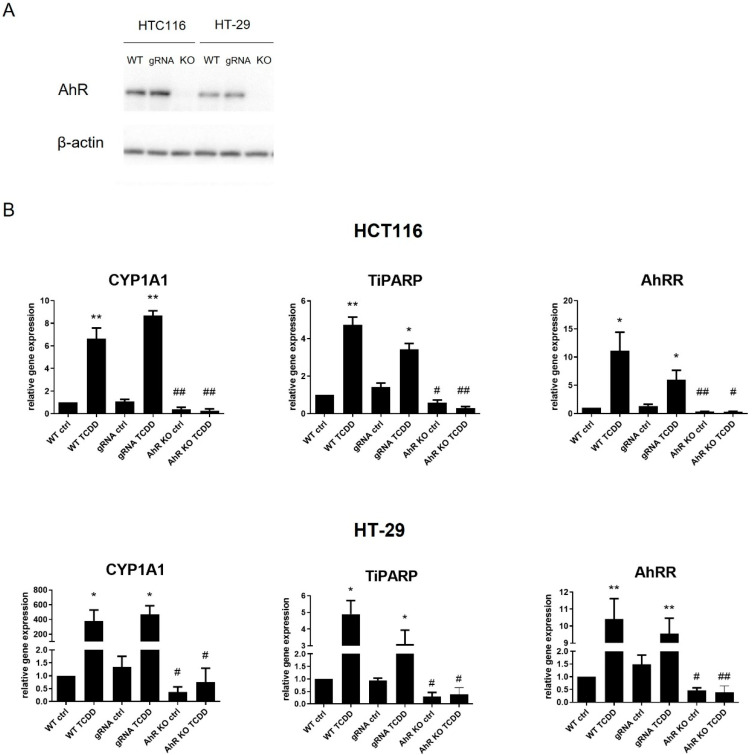
Loss of the AhR blocks induction of its target genes in HT-29 and HCT116 AhR KO cells. (**A**) Wild-type cells (WT), cells transfected with a CRISPR-Cas9 empty vector (gRNA) and AhR KO cell clones of HCT116 and HT-29 were used for detection of the AhR protein levels by Western blotting. For original blots, see Supplementary File S1. (**B**) Induction of AhR-target gene mRNAs after 24 h treatment with TCDD (10 nM) was determined by RT-qPCR. The data are shown as means + SD of at least three independent experiments. * and ** denote a significant difference (*p* < 0.05 and *p* < 0.01, respectively) between TCDD-treated and respective control cells. # and ## denote a significant difference (*p* < 0.05 and *p* < 0.01, respectively) between TCDD-treated AhR KO cells and the respective TCDD-treated wild-type cells.

In order to analyze the potential role of the AhR in control of colon carcinoma cell behavior, we employed the AhR KO clones derived from parental HT-29 and HCT116 cells lines by CRISPR/Cas9 technique. We first confirmed that neither of the KO variants expressed the AhR protein (Figure 2A) and that there was no induction of expression of three principal AhR gene targets (*CYP1A1*, *TIPARP* and *AHRR*) in the AhR KO cells treated with a potent AhR agonist, TCDD (Figure 2B). We then compared cellular morphology of AhR KO cells with the parental cell lines. Using cell painting analysis (a high-content, multiplexed image-based assay used for cytological profiling), we found no significant differences between wild-type and corresponding AhR KO cells in any of the traits studied (Supplementary Materials, Figure S1). This indicated that the loss of the AhR was not associated with gross morphology alterations in either the HT-29 or HCT116 cell lines.

**Figure 3:**
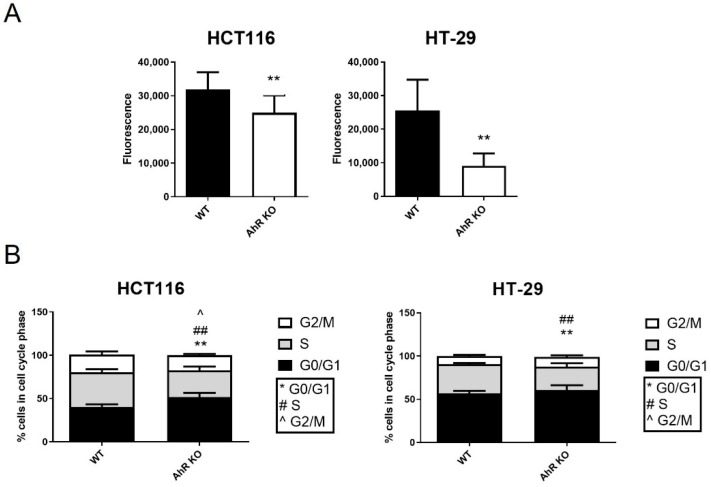
Loss of AhR reduces proliferative rate of HCT116 and HT-29 cells. Cell numbers (**A**) and cell cycle distribution (**B**) of wild-type cells (WT) and AhR KO clones were determined 72 h after seeding by CyQUANT assay and flow cytometric analysis of cell cycle, respectively. The data are shown as means + SD of at least three independent experiments. ** denotes a significant difference (*p* < 0.01) between G0/G1 S-phase numbers of WT and AhR KO cells; ^##^ denotes a significant difference (*p* < 0.01) between S-phase numbers of WT and AhR KO cells; ^ denotes a significant difference (*p* < 0.05) between G2/M numbers of WT and AhR KO cells.

We then evaluated potential impact of the AhR deficiency on proliferative behavior of colon carcinoma HT-29 and HCT116 cells lines. Using DNA content quantification by CyQUANT assay, we found that AhR KO clones have a decreased proliferation rate, as compared with the respective wild-type cell line (Figure 3A). The reduced proliferative rate was confirmed also by counting cell numbers in HCT116 AhR KO cells (Supplementary Materials, Figure S2). We further analyzed cell cycle phase distribution by flow cytometry at the same time point (72 h). We observed a decreased percentage of cells in S-phase and an increased percentage of cells in G0/G1 phase of cell cycle, both in HCT116 and in HT-29 AhR KO cells, as compared with the respective parental cell line (Figure 3B). The percentage of cells in G2/M phase was significantly lower only in HCT116 KO cells (Figure 3B). 

Since the Wnt/β-catenin pathway is considered to be a major regulator of the proliferation of colon cancer cells, and the AhR has been proposed to interfere with β-catenin-dependent signaling in colon cells [20], we also analyzed the levels of β-catenin in wild-type and AhR KO HCT116 cells. Since β-catenin levels can be influenced by cell density, we used cells cultured at different confluency levels for this analysis. We observed no differences in either total or active β-catenin when we compared parental and AhR KO HCT116 cells (Supplementary Materials, Figure S2B). In addition, there were no differences observed between basal or induced Wnt/β-catenin pathway activity, as determined by TOP/FOP assay (Supplementary Materials, Figure S2C).

### 3.3. AhR Deficiency Alters Cellular Energy Metabolism in HCT116 and HT-29 Cells 

**Figure 4:**
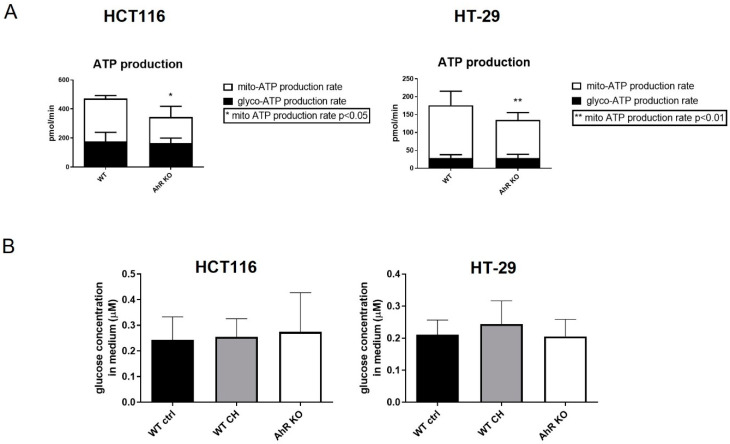
AhR deficiency alters ATP production rate in HCT116 and HT-29 cells. (**A**) Real-time ATP production rate was measured by SeaHorse XFp, and the contribution of mitochondria-produced ATP (mito-ATP) and glycolysis-produced ATP (glyco-ATP) to total ATP levels was determined. The data are shown as means + SD of three independent experiments. * and ** denote a significant difference (*p* < 0.05 and *p* < 0.01, respectively) between AhR KO and wild-type cells. (**B**) Glucose uptake was measured by colorimetric assay determining the concentration of glucose in cell culture medium collected from cells after 48 h of cultivation. The effect of AhR KO was compared with that of incubation of cells with the AhR antagonist CH-223191 (10 μM).

Next, we determined the rate of ATP production and estimated relative contribution of glycolysis and mitochondrial oxidative phosphorylation to ATP production, by using real-time measurement of the oxygen consumption rate and the extracellular acidification rate by the SeaHorse XFp analyzer. The total ATP production rate was significantly lower in AhR KO HCT116 and HT-29 cells, as compared with wild-type cells (Supplementary Materials, Figure S3). We found that this was primarily due to reduced mitochondrial production of ATP (mito-ATP), while production of glycolysis-derived ATP (glyco-ATP) was not altered in AhR KO cells (Figure 4A). In order to examine whether the reduction of ATP production rate could be linked with altered glucose consumption, we also determined levels of glucose in media collected from AhR KO and wild-type cells. The glucose uptake was not changed in any of cell lines studied (Figure 4B). In addition, exposure to the AhR antagonist, CH-223191, did not impact glucose consumption in either HCT116 or HT-29 cells (Figure 4B).

### 3.4. Loss of the AhR Affects Expression of FA Synthesis Genes and Limits Akt Signaling

**Figure 5:**
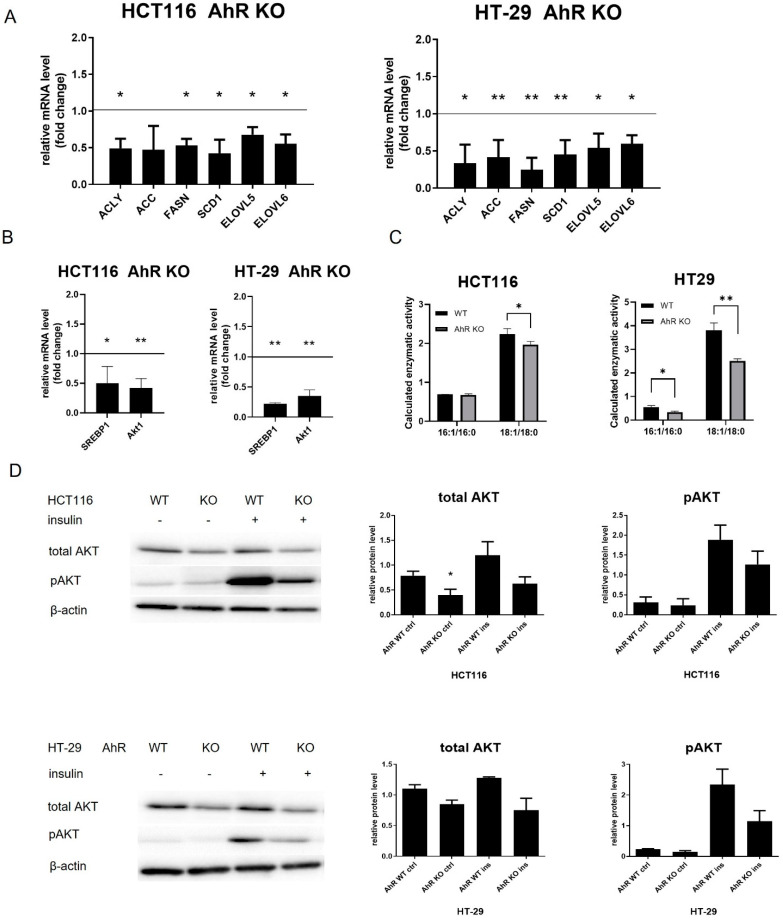
Loss of the AhR reduces expression of FA synthesis genes and their regulators, alters calculated SCD1 activities and modulates Akt protein levels/phosphorylation. The levels of mRNAs encoded by genes involved in FA synthesis or control of lipid metabolism 48 h after their seeding (**A**,**B**). The data are shown as means + SD of at least three independent experiments. * and ** denote a significant difference (*p* < 0.05 and *p* < 0.01, respectively) between AhR KO and wild-type cells. The full line indicates mRNA level in wild-type cells. (**C**) The levels of palmitoleic (C16:1), palmitic (C16:0), oleic (C18:1) and stearic (C18:0) fatty acids in wild-type (WT) and AhR KO cell lines were determined by GC/MS, and ratios of C16:1/C16:0 and C18:1/18:0 were calculated. The data are shown as means + SD of three independent experiments. * and ** denote a significant difference (*p* < 0.05 and *p* < 0.01, respectively) between AhR KO and wild-type (WT) cells. (**D**) Western blotting analysis of levels of total Akt and phosphorylated Akt (pAkt; Ser473) with or without treatment by insulin (ins; 100 nM) for 1 h. Representative Western blot images are shown at the left; results of densitometry analysis, normalized to β-actin, are shown at the right. For original blots, see Supplementary File S1. The data are shown as means + SD of at least three independent experiments. * denotes a significant difference (*p* < 0.05) between AhR KO and wild-type (WT) cells.

FAs represent important sources of energy. They are crucial for sustaining a high proliferation rate in cancer cells, but they are also important building blocks of cell membranes [56]. Therefore, we next evaluated the impact of the AhR loss on mRNA levels of key enzymes involved in de novo synthesis of FAs: acetyl-CoA lyase (ACLY), ACC, FASN, and SCD1, as well as FA elongases 5 and 6 (ELOVL5, ELOVL6). Both AhR KO HCT116 and HT-29 cells showed reduced levels of FA synthesis, desaturation and elongation enzymes (Figure 5A). The mRNA levels of the master regulator of expression of genes involved in de novo synthesis of FAs, transcription factor sterol regulatory element binding transcription factor 1 (SREBP1), were also lower in AhR KO cells (Figure 5B). We then performed analyses of lipid and FA content in wild-type and AhR KO cells. We did not observe significant changes in total neutral lipid or phospholipid content between wild-type and AhR KO cells (Supplementary Materials, Figure S4A). Total levels of FAs (Supplementary Materials, Figure S4B), or sums of saturated, monounsaturated or polyunsaturated FAs (Supplementary Materials, Figure S4B) were also not altered in AhR KO cells. Nevertheless, when we calculated C16:1/16:0 and C18:1/18:0 ratios reflecting enzymatic activity of SCD1, we found a small albeit significant decrease in both ratios in HT-29 AhR KO cells, and in C18:1/18:0 ratio in HCT116 AhR KO cells (Figure 5C).

We observed that levels of Akt1 isoform (*AKT1*), a part of the Akt pathway, which controls many aspects of cellular metabolism, were significantly lower in AhR KO HCT116 and HT-29 cells (Figure 5B). The expression levels of the other two Akt isoforms, *AKT2* and *AKT3*, were also altered in AhR KO cells, but in a cell-dependent manner: Akt2 mRNA was lower only in HT-29 AhR KO cells, while levels of Akt3 mRNA were significantly lower in HCT116 cells (Supplementary Materials, Figure S5A). We next examined total Akt protein levels, as well as both basal and insulin-induced Akt phosphorylation in AhR KO cells. We found that HCT-116 AhR KO cell lines exhibit significantly lower levels of total Akt protein, and a similar trend, albeit not significant, was observed in HT-29 AhR KO cells (Figure 5D). The treatment with insulin, which is known to activate the Akt pathway [57], has been used as a model activator of Akt signaling. Both AhR KO cell lines exhibited a trend toward reduced levels of Ser473-phosphorylated Akt (Figure 5C). 

Phosphatase and tensin homolog (PTEN) is a phosphatase acting as a negative regulator of Akt phosphorylation, which blocks Akt dependent signaling, thus reducing transcription of Akt target genes. We used the HCT116 PTEN KO cell line in order to confirm that Akt activity is directly linked with control of lipogenic genes in HCT116 cells. We confirmed that expression of ACLY, ACC, FASN, SCD1, as well as that of AKT1 and SREBP1 is higher in PTEN KO cells (Supplementary Materials, Figure S5B). 

### 3.5. AhR Antagonist CH-223191 Inhibits Cell Proliferation and Expression of FA Synthesis-Related Genes and Their Regulators in Colon Cancer Cells

**Figure 6:**
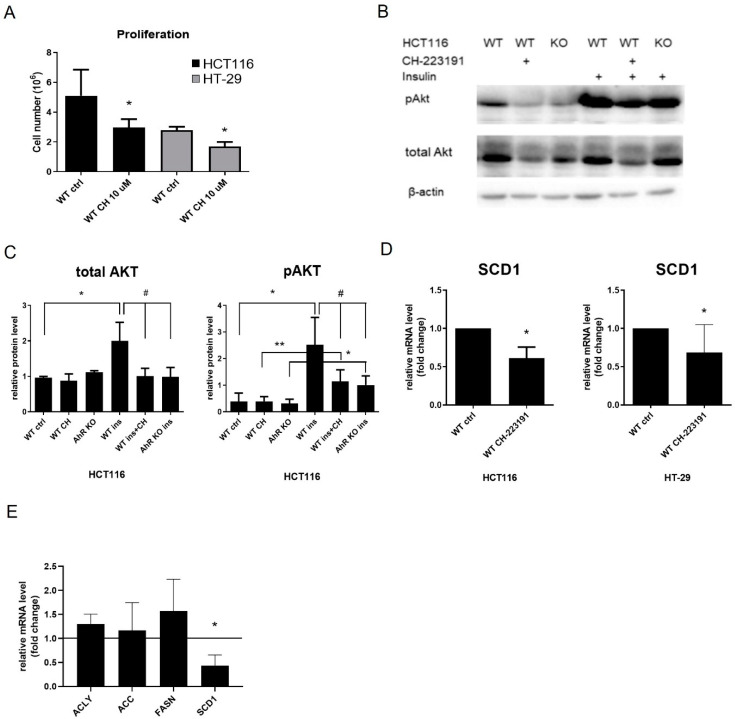
The AhR antagonist CH-223191 interferes with proliferation, Akt pathway activity and SCD1 expression in colon carcinoma cells. (**A**) Cells were treated with CH-223191 (10 μM), 24 h after their seeding, and cell numbers were determined after 48 h treatment. The data are shown as means + SD of at least three independent experiments. * denotes a significant difference (*p* < 0.05) between CH-223191-treated and control cells. (**B**,**C**) HCT116 cells were pre-treated with CH-223191 (10 μM) for 48 h and then with insulin (100 nM) for 1 h. The levels of phosphorylated Akt (pAkt; Ser473) and total Akt were determined by Western blotting, with β-actin being used as loading control. Representative Western blot images are shown in (**B**). For original blots, see Supplementary File S1; the results of densitometry analysis, normalized to β-actin are shown in (**C**). The data are shown as means + SD of at least three independent experiments. * and ** denote a significant difference (*p* < 0.05 and *p* < 0.01, respectively) between insulin-treated and the respective control cells. # denotes a significant difference (*p* < 0.05) between control wild-type cells treated with insulin and insulin treated AhR KO cells or insulin-treated wild-type cells pre-treated with CH-223191. (**D**) SCD1 mRNA levels were determined in control HT-29 and HCT116 cells, and in cells treated with CH-223191 (10 μM) for 24 h, by RT-qPCR. The data are shown as means + SD of at least three independent experiments. * denotes a significant difference (*p* < 0.05) between control and CH-223191-treated cells. (**E**) Expression of FA synthesis/transport genes in WT (line) and AhR KO differentiated HepaRG cells (black bars). The data are shown as means + SD of at least three independent experiments. * denotes a significant difference (*p* < 0.05) between WT and AhR KO cells.

In order to further verify our observations of the impact of the AhR loss on cell proliferation and expression of FA synthesis genes in colon cancer cell lines, we next used the AhR antagonist CH-223191, in order to inhibit the AhR signaling. Following 48 h treatment with CH-223191, both HCT116 and HT-29 cells had lower cell numbers than control cells (Figure 6A). CH-223191 also reduced levels of total Akt in a similar manner as the loss of the AhR in HCT116, and it inhibited Akt phosphorylation upon treatment of HCT116 cells with insulin (Figure 6B,C). Based on this, we also analyzed expression of SCD1, which has been proposed to be directly regulated by the AhR [44]. In both HCT116 and HT-29 cell lines, the AhR antagonist induced a decrease in SCD1 mRNA (Figure 6D). The mRNA levels of other FA synthesis enzymes and Akt1 were also significantly lower in HCT116 cells treated with another AhR antagonist, StemRegenin 1 (Supplementary Materials, Figure S6). 

Finally, in order to determine the impact of AhR knockout on expression of FA metabolism genes in an independent model of non-proliferating differentiated cells, we employed differentiated hepatocyte-like HepaRG cells. Here, we found that the AhR loss reduced levels of SCD1 mRNA, but not the expression of other FA synthesis/transport genes (Figure 6E). This indicated that whereas SCD1 could be controlled directly by the AhR activity, the effects on other FA synthesis and regulatory genes could be linked to the impact of AhR on cell proliferation and metabolism in colon cancer cells. 

## 4. Discussion

The overexpression of AhR and/or its increased activity has been observed in a number of tumor types, including breast cancer, lung adenocarcinoma, gastric cancer, adult T-cell leukemia, pancreatic cancer, prostate cancer, urothelial carcinoma, human glioma or medulloblastoma [58]. Several reports have indicated that AhR mRNA and/or AhR protein can be upregulated in colon tumor tissue [23,24,48,59]. Importantly, in the present study, we found that increased levels of AhR protein can be found not only in whole colon tumor tissue, but directly in EpCAM^+^ epithelial cells isolated from CRC tumors. Collectively, these observations have led us to explore the functional role of the AhR, using full CRISPR/Cas9-mediated knockout of the AhR in two models of colon carcinoma cells, HCT116 and HT-29 cell lines. These two cell lines represent two types of colon cancer cell lines, one that is able to differentiate (HT-29 cells), and HCT116 cells, which do not differentiate in vitro and which represent advanced colon carcinoma model capable of forming high-grade tumors and metastases in vivo [60]. We focused on the impact of the AhR loss on cell proliferation and metabolism, in particular on the pathways contributing to increased production of FAs, given our previous observation that upregulation of the AhR may occur in parallel with a deregulation of FA, sphingolipid and phospholipid metabolism in colon cancer cells [47,48].

The activation of the AhR with its strong ligands has been shown to elicit cell cycle perturbations [34]. It has been reported that AhR knockdown mediated by siRNA can increase proliferation of colon cancer cell lines [23]. However, in the present study, we observed that a full AhR KO decreased cell proliferation in these cellular models. Clonal variability in colon cancer cell lines modified via CRISPR/Cas9 should be considered when interpreting our data. Nevertheless, the observed discrepancy could also be partly explained by different impacts of siRNA-mediated knockdown and full absence of AhR gene in cells, which has been observed for other genes [61]. For example, even low levels of the AhR could be affected by endogenous ligands of the AhR derived from tryptophan being present in cell culture media [62]. It cannot be excluded that some of the differences observed between wild-type and AhR KO cells in the present study could be due to tryptophan metabolites present in the cell culture medium, which may weakly activate the AhR in wild-type cells, as various types of tryptophan metabolites have been shown to modulate functions of intestinal cells [49,63].

Together with a decreased proliferative rate, the AhR KO cells also exhibited alterations in cell cycle progression: a decreased percentage of cells in S-phase; and an increased percentage of cells in G0/G1 phase. In the cells without the AhR, a slower cell cycle progression has been observed, which can be linked to the function of the AhR in the promotion of cell cycle progression in the absence of an exogenous ligand [34]. This seems to support the present observations. The absence of AhR in mouse hepatoma cells or its knockdown in human hepatoblastoma cells leads to a delay in the G1 to S-phase transition [64,65]. Activation of the AhR by its powerful agonist, TCDD, may also inhibit cell proliferation and induce cell cycle arrest [31]. This can be mediated by increased expression of the p27^Kip1^ cyclin-dependent kinase inhibitor or via further interactions of the AhR with Rb protein [66]. The relationship between the AhR and cell cycle regulators can be bi-directional, as both Rb and p27^Kip1^ have been shown to regulate the AhR-mediated transcription [67,68]. Here, we found that the AhR antagonist CH-223191 inhibited the proliferation of HT-29 and HCT116 cells (in a similar manner as AhR KO), which seems to provide further support to the hypothesis that the AhR plays a positive role in the control of colon cancer cell proliferation, perhaps in a similar manner as in the above discussed models of liver carcinoma cells. The Wnt/β-catenin signaling pathway is an important regulator of proliferation of colon cancer cells, and it has been proposed to be inhibited via the AhR-mediated degradation of β-catenin in colon epithelial cells [20]. Nevertheless, other studies did not observe the AhR agonist-mediated degradation of β-catenin in human colon cancer cell models [69]. This, together with our observation that the levels of total/active β-catenin and activity of the TCF/LEF reporter were not affected by the AhR loss in HCT116 cells, indicates that the potential role of the AhR in control of proliferation of colon cancer cells may not depend on β-catenin activity.

The lower proliferative rate of HT-29 and HCT116 AhR KO cells was accompanied with further disturbances of cellular metabolism. In both cells lines, the rate of mitochondrial ATP production was lower than in the matching wild-type controls, although we observed no differences in glucose consumption. The AhR plays a role in the control of body energetic metabolism, and the use of AhR antagonists has been reported to reduce obesity, adiposity or liver steatosis in mice fed with Western diet [70,71]. The AhR has been also reported to participate in the control of metabolic pathways, which contribute to the proliferation of tumor cells [40]. Furthermore, TCDD has been shown to decrease mitochondrial respiration in mouse hepatoma cells, and the basal oxygen consumption rate appears to be lower in AhR-deficient cells [72], which seems to support our observations. Nevertheless, it also cannot be excluded that the impact of AhR loss on mitochondrial ATP production was linked to the cell cycle disturbances observed in the AhR KO cells. In general, the altered proliferative rate (which is known to impact cell metabolism) of the AhR KO cells should be kept in mind when interpreting the effects of the AhR KO on lipid metabolism observed in the present study. 

One of a key metabolic changes occurring during malignant transformation is activation of de novo synthesis of FAs and lipids, which contributes to cancer development and progression [73]. Cancer cells become less dependent on extracellular supplies and can satisfy their high lipid demand for sustaining rapid proliferation [74]. When assessing mRNA levels of lipid synthesis-related genes, we found ACC, ACLY, FASN and SCD1, which catalyze key steps in FA synthesis, to be decreased in both HCT116 and HT-29 AhR KO cells. In addition, two FA elongases, ELOVL5 and 6, were also downregulated in AhR KO colon cancer cells. Thus, in addition to decreased cell proliferation and ATP production, AhR deficiency may also lead to deregulation of FA synthesis in colon cancer cells. However, we did not observe major alterations of lipid or FA content in the AhR KO cells, which could be linked to the fact that the levels of monounsaturated and polyunsaturated FA levels in cancer cells cultured in vitro can be significantly affected by the fetal calf serum present in cell culture medium [75]. Nevertheless, we found the C18:1/18:0 ratio to be lower in both AhR KO models and C16:1/16:0 ratio to be lower in HT-29 AhR KO cells. This indicated that the observed changes in SCD1 expression could be linked to a partly reduced production of some monounsaturated FAs. 

Previously, TCDD has been reported to regulate expression of the *Scd1* gene in mouse liver [44,76], and numerous other toxic AhR agonists, including benzo[a]pyrene, 2,3,7,8-tetrachlorodibenzofuran or 3,3′,4′,4′,5-pentachlorobiphenyl, have been also shown to markedly alter lipid composition in the mouse liver [77,78,79]. Our present data indicate that both in colon cancer cells and in non-proliferating differentiated human liver HepaRG cells, the AhR deficiency leads to downregulation of SCD1 mRNA, suggesting that the AhR contributes to its transcriptional control in a manner that is independent of cell cycle deregulation. The SCD1 levels were also significantly reduced by two chemical AhR antagonists (CH-223191 and StemRegenin 1) in colon cancer cells. The deregulation of colon cancer lipid metabolism has been shown to correlate with poor prognosis in CRC patients [80]. SCD1 is a key enzyme converting saturated FAs into monounsaturated FAs, and its inhibition has been shown to lead to cancer cell death [81]. It promotes migratory and invasive properties through epithelial–mesenchymal transition (EMT) induction, and it may increase the risk of relapse in CRC patients [82]. As SCD1 plays vital roles in CRC and in colon cancer cells [83,84], the potential role of the AhR in control of SCD1 expression in colon tumor cells deserves more attention. Importantly, SCD1 was the only FA synthesis enzyme found to be downregulated in the AhR KO liver HepaRG cells, a cell model representing slowly proliferating differentiated cells. This indicates that inhibition of SCD1 expression in the absence of the AhR is not linked to reduced cell proliferation observed in AhR KO colon cancer cells.

One of the principle regulators of cellular metabolism is the phosphoinositide 3-kinase (PI3K)/Akt pathway. It participates in the control of metabolism of glucose, biosynthesis of lipids, proteins and nucleotides, as well as in the regulation of cellular redox homeostasis [85]. Constitutive activation of the Akt pathway leads to an increased expression of lipid synthesis-related genes, including their transcriptional regulators, such as SREBP1 [86]. When we compared Akt expression (levels of three Akt isoform mRNAs and total protein), we found significantly reduced mRNA and protein levels of Akt in AhR KO cells. Moreover, following the activation of PI3K with insulin, we observed lower levels of Ser473-phosphorylated Akt in AhR KO clones than in wild-type cells. Presently, the role of the AhR in the control of Akt signaling is not fully clear. Lack of the AhR has been shown to be associated with an impaired activation of Akt in mouse hepatoma cells [87]. In contrast, phosphorylation of Akt is higher in the livers of AhR KO mice [38]. It has been also reported that TCDD (via an AhR-dependent mechanism) regulates the Akt pathway in breast cancer stem cells through inhibition of PTEN phosphatase [37]. When using HCT116 PTEN-deficient cells, we observed upregulation of similar targets, which were inhibited in AhR KO cells. However, we found no changes in PTEN protein levels in AhR KO cells (data not shown), which suggests that the observed effects were not due to PTEN deregulation. It is possible that the impact of AhR on Akt may depend on additional factors controlling PI3K/Akt pathway activity, including the reduced proliferation of the AhR KO cells. Nevertheless, the data obtained with AhR KO clones were further verified by using chemical inhibition of the AhR. We found that, in addition to decreasing cell numbers in colon cancer cells, the AhR antagonist CH-223191 significantly impaired activation of the Akt pathway. It reduced total Akt protein levels, and it inhibited phosphorylation of Akt at Ser473 in insulin-treated HCT116 cells, which mimicked the impact of the AhR loss. Together, these data indicate that aberrant expression of the AhR in colon cancer cells might also promote Akt pathway activity, with further implications for deregulation of cell growth, survival and metabolism, including FA metabolism.

## 5. Conclusions

In the present study, we employed AhR KO models of two colon carcinoma cell lines in order to better understand the impact of this transcription factor, which is frequently overexpressed in colon tumors, on their proliferative and metabolic behavior. Our results show that the loss of AhR or its chemical inhibition may lead to the disruption of growth and metabolism of colon cancer cells, when cultured under standard in vitro conditions. AhR deficiency led to disruption of mitochondrial ATP production, as well as to alterations of expression of several genes involved in FA synthesis. This was accompanied with reduced Akt expression/phosphorylation, and the downregulation of a key lipogenic transcription factor SREBP1. While some of these effects could be linked with the anti-proliferative consequences of the AhR knockout, the present results also indicate that some FA metabolism genes, in particular SCD1, might be regulated by the AhR in colon cancer cells. Future studies should extend our findings to a larger panel of colon cancer cell models, including ex vivo and in vivo approaches, in order to further develop these observations, as many aspects of the potential role of AhR in the control of proliferation of colon tumor cells, their tumorigenicity or metabolic features remain only partially understood. A better understanding of these processes may provide further background for exploration of lipid metabolism as a metabolic vulnerability in colon cancer.

## Data Availability

The data presented in this study are available on request from the corresponding author.

## Supplementary Materials

The following are available online at https://www.mdpi.com/article/10.3390/cancers14174245/s1: Figure S1: Cell morphology analyses; Figure S2: HCT116 AhR KO cells proliferate slower, but activity of Wnt/β-catenin pathway is not affected; Figure S3: Total ATP production in colon cancer wild-type and AhR KO cells; Figure S4: Effects of AhR KO on total lipid and FA content in colon cancer cell lines; Figure S5: Supporting RT-qPCR analyses; Figure S6: RT-qPCR of FA synthesis genes in HCT116 cells treated with StemRegenin1; Table S1. List of primers and probes used for RT-qPCR; Table S2: List of antibodies used for Western blotting. File S1: Original blots of Figures 1D, 2A, 5D, 6D and S2.
