# Supplementary material for: Inhibition of Aryl Hydrocarbon Receptor (AhR) Expression Disrupts Cell Proliferation and Alters Energy Metabolism and Fatty Acid Synthesis in Colon Cancer Cells
Source: Cancers (Basel). 2022 Aug 31;14(17):4245. doi: 10.3390/cancers14174245 (PMC9454859; doi:10.3390/cancers14174245)
Supplement: Supplementary file 1 [file cancers-14-04245-s001.zip › cancers-1864999-supplementary tables and figures.pdf]

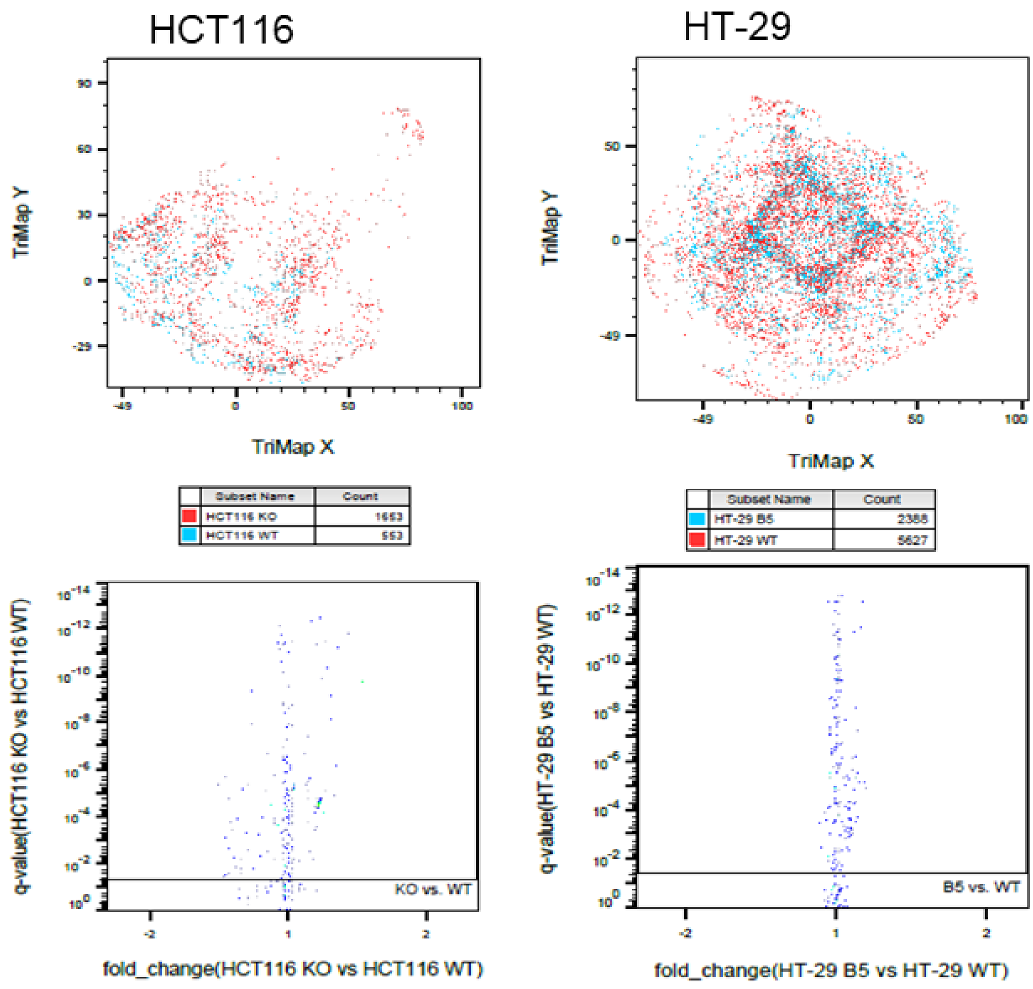

Figure S1. Cell painting morphology analysis. For cell painting morphology analysis the cells were stained with Hoechst 33342, concanavalin A, SYTO14, phalloidin, wheat germ agglutinin and MitoTracker. Images were acquired with using ImageXpress Micro (Molecular Devices) fluorescence microscope (40x objective). Objects of nuclei, cytoplasm and whole cell areas were segmented and together 1785 features were calculated from 5 fluorescence channels on these three objects with using CellProfiler v2.2.0 software. Principal component analysis and t-SNE analysis (perplexity 1605, nr. of iterations 500, minimum cost value 0.5) were performed. The above plots were produced in R.

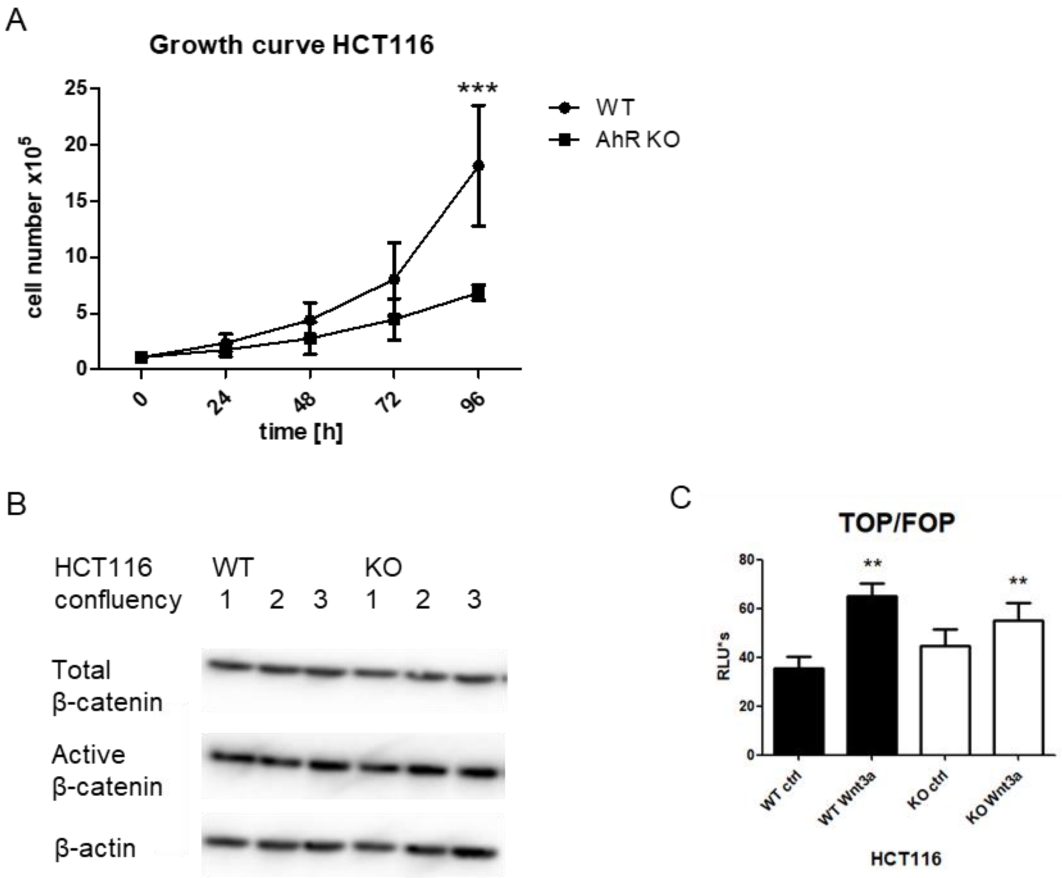

Figure S2. HCT116 AhR KO cells proliferate slower, but activity of Wnt/β-catenin pathway is not affected**.** A) Wild-type and AhR KO cells were grown under standard conditions, and counted every 24 h. Data represent means + SD of at least three independent experiments. The difference between WT and AhR KO cells was estimated by two-way ANOVA. The statistical significance: * $p<0.05$, *** $p<0.001$. B) HCT116 cell line was used for expression analysis of β-catenin (total and active). Cells were grown at three different confluence levels (level 1 < 50%; level 2: 70-80%; level 3 > 90%). β-catenin levels (both total and active) were detected by Western blotting. β-actin levels were used as loading control. C) Wnt-pathway activity was measured by TOP/FOP luciferase assay. Data represent means + SD of three independent experiments. The statistical significance: ** $p<0.01$.

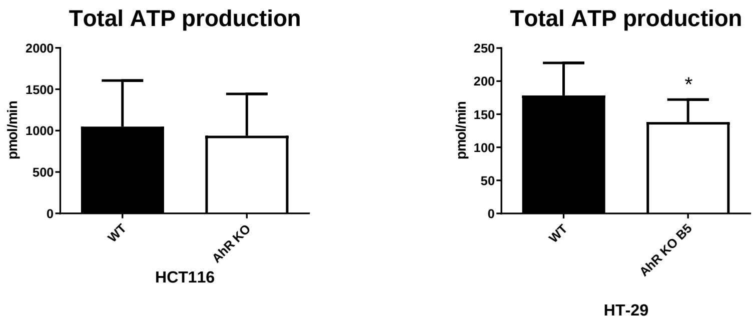

Figure S3. Total ATP production in colon cancer wild type and AhR KO cells. The rate of ATP production was assessed by SeaHorse XFp, using the XFp real-time ATP rate assay kit. Data represent means + SD of at least three independent experiments. The statistical significance: * $p<0.05$, ** $p<0.01$.

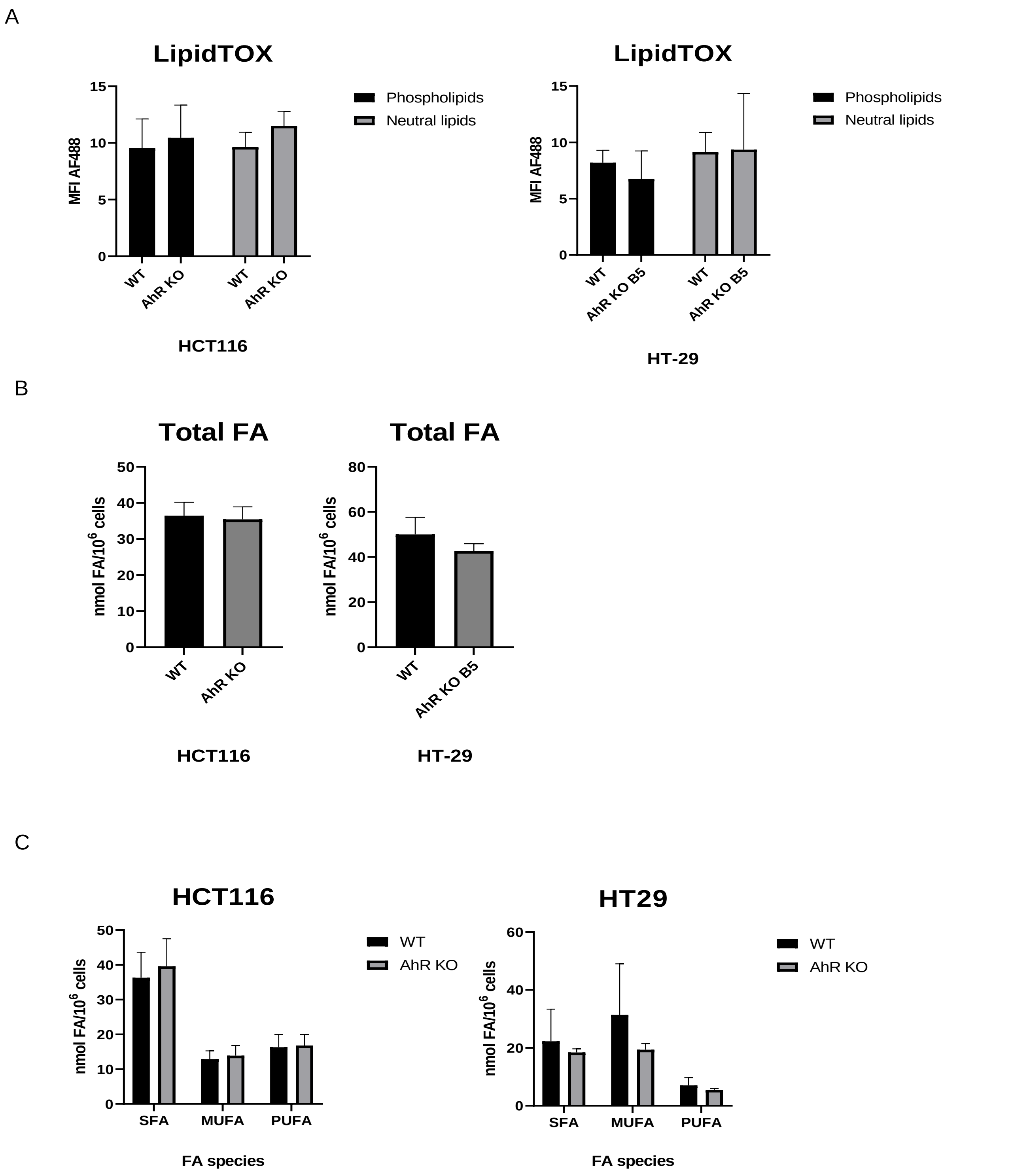

Figure S4. Lipid and fatty acid analyses. (A) Total levels of neutral lipids and phospholipids in cells were estimated by LipidTOX staining, using flow cytometry. Data represent means + S.D. of three independent experiments. (B,C) Based on analysis of fatty acids described in the main body of the text, total levels of saturated (SFA), monounsaturated (MUFA) and polyunsaturated (PUFA) fatty acids were calculated. Data represent means + S.D. of three independent experiments.

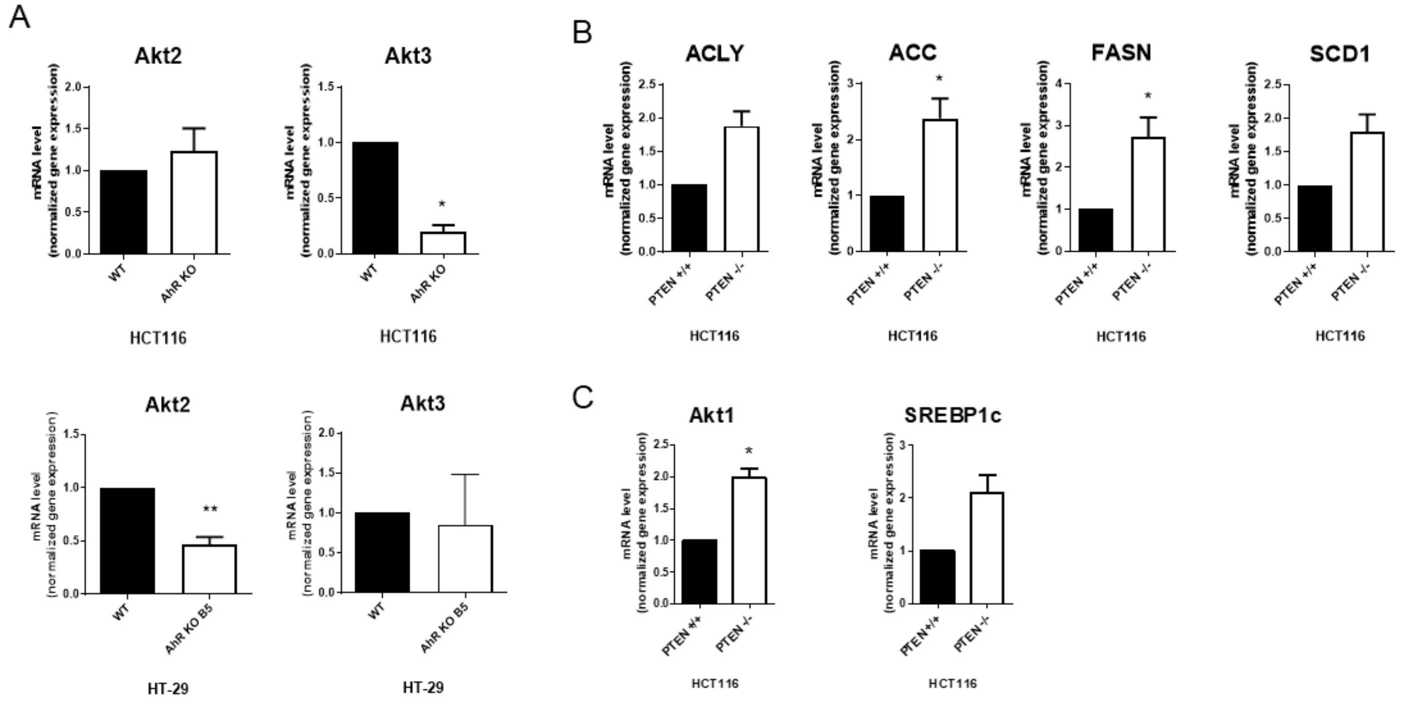

Figure S5. Supporting RT-qPCR analyses**.** A) RT-qPCR analysis of mRNA levels of Akt 2 and Akt3 in wild-type HCT116 and HT-29, and in their WT and AhR KO cells, respectively. B) RT-qPCR analysis of gene expression in PTEN-deficient HCT116 cell line and wild-type HCT116 cells (B,C). Data represent means of at least three independent experiments. The statistical significance * p<0.05, ** p<0.01.

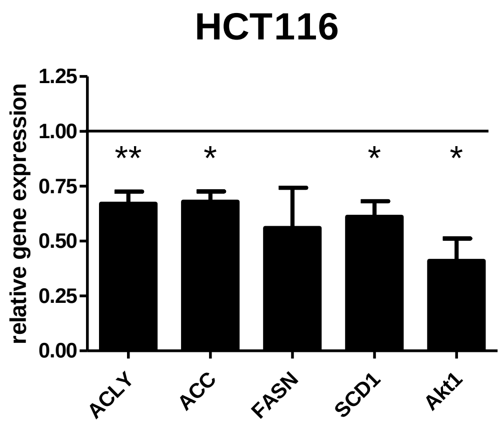

Figure S6. RT-qPCR of FA synthesis related genes in HCT116 cells treated with StemRegenin1. Cells were treated with StemRegenin1 (1 μM) for 48 h, or with DMSO a s a solvent control. Data represent means of at least three independent experiments. The statistical significance * p<0.05, ** p<0.01.

Table S1. List of primers and probes used for RT-qPCR.

Gene Card ID	Name / abbreviation	Sequences 5'-3'	
GC15M074719	Cytochrome P450 Family 1 Subfamily A Member 1	forward	caccatcccccacagcac
	CYP1A1	reverse	ttacaaagacacaacgcccc
		probe	caagtttgaaaggcttttacatcccc
GC03P156673	TCDD Inducible Poly(ADP-Ribose) Polymerase	forward	ggaaattcttctgtagggacca
	TiPARP	reverse	aatcaatcgaatgacagactcg
		probe *	58
GC05P000321	Aryl-Hydrocarbon Receptor Repressor	forward	gcaaaacccagagcagacac
	AhRR	reverse	acagactggtggtggctttta
		probe *	83
GC17M082078	Fatty Acid Synthase	forward	caggcacacacgatggac
	FASN	reverse	cggagtgaatctgggttgat
		probe *	11
GC17M041866	ATP Citrate Lyase	forward	aaccccaaagggaggatct
	ACLY	reverse	ttgacacccccctagatcacag
		probe *	49
GC17M037084	Acetyl-CoA-carboxylase alfa	forward	cattccgagcaagggataag
	ACC	reverse	cagagcaggctccagatga
		probe *	101
GC10P100347	Stearoyl-CoA Desaturase	forward	cctagaagctgagaaactggtga
	SCD	reverse	acatcatcagcaagccaggt
		probe *	87
GC14M104769	AKT Serine/Threonine Kinase 1	forward	ggctattgtgaaggagggttg
	Akt1	reverse	tccttgtagccaatgaaggtg
		probe *	69
GC19M040230	AKT Serine/Threonine Kinase 2	forward	ctcacacagtcaccgagagc
	Akt2	reverse	tgggtctggaaggcatactt
		probe *	65
GC01M243488	AKT Serine/Threonine Kinase 3	forward	ttgctttcagggctcttgat
	Akt3	reverse	cataatttcttttgcatcatctgg
		probe *	22
GC17M017810	Sterol Regulatory Element Binding Transcription Factor 1c	forward	cgctcctccatcaatgaca
	SREBF-1c	reverse	tgcgcaagacagcagattta
		probe *	77
GC06M053267	ELOVL Fatty Acid Elongase 5	forward	cccttccatgcgtccata
	ELOVL5	reverse	tgtcagcacaaactgaagcag
		probe *	31
GC04M110045	ELOVL Fatty Acid Elongase 6	forward	caaagcacccgaactaggag
	ELOVL6	reverse	tggtgataccagtgcaggaa
		probe *	38

GC06P170554	TATA-Box Binding Protein	forward	gaacatcatggatcagaacaaca
	TBP	reverse	atagggattccgggagtcat
		probe *	87

* the number of Universal Probe Library (Roche Diagnostics) probe used.

Table S2. List of antibodies used for Western blotting analyses.

**Protein**	**Brand**	**cat. N.**	**dilution**
β-actin	Sigma-Aldrich	A5441	1:5000
AhR	Cell Signaling Technology	cs-83200	1:500
AhR	Thermo Fisher Scientific	MA1-514	1:500
Akt	Cell Signaling Technology	cs-9272	1:1000
Phospho-Akt (Ser473)	Cell Signaling Technology	cs-9271	1:100
β-catenin	Cell Signaling Technology	cs-8480	1:1000
Active β-catenin (ABC)	Cell Signaling Technology	cs-9561	1:1000
