# Supplementary material for: Inhibition of Aryl Hydrocarbon Receptor (AhR) Expression Disrupts Cell Proliferation and Alters Energy Metabolism and Fatty Acid Synthesis in Colon Cancer Cells
Source: Cancers (Basel). 2022 Aug 31;14(17):4245. doi: 10.3390/cancers14174245 (PMC9454859; doi:10.3390/cancers14174245)
Supplement: Supplementary file 1 [file cancers-14-04245-s001.zip › File S1-Western blot image.pdf]

Figure 1D

AhR

β-actin

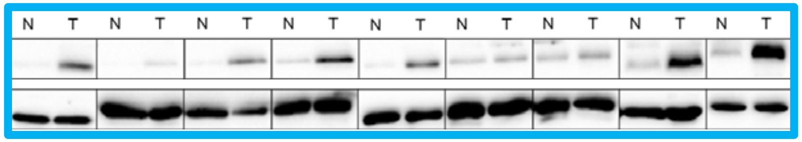

2,0	7,5	1,6	2,3	1,7	5,7	2,3	9,4	1,6	6,7	3,1	4,1	4,1	4,9	4,4	15,9
3,7	3,8	7,7	5,7	5,5	3,7	6,0	7,5	5,4	4,7	7,1	7,3	6,3	6,4	5,3	5,8

% peak area

AhR

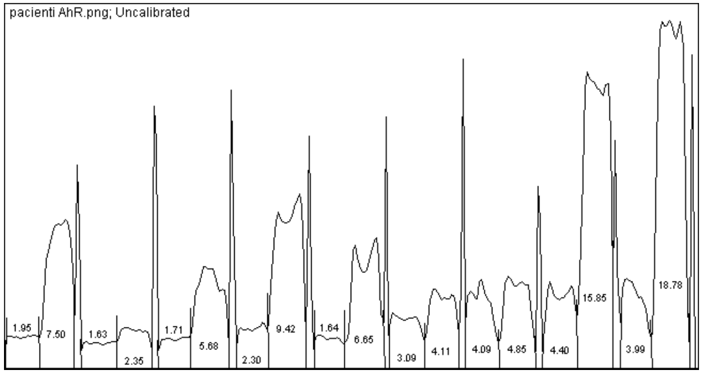

β-actin

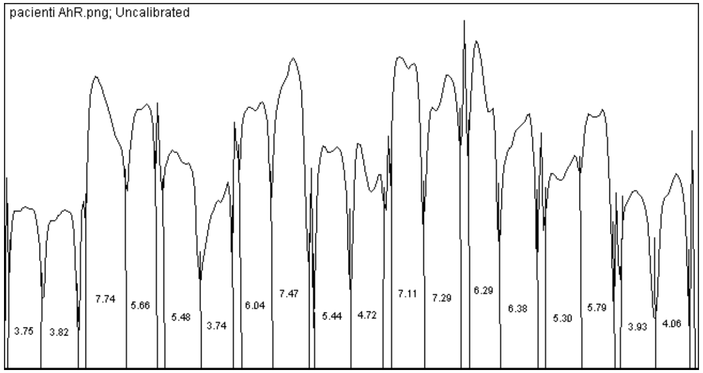

Figure 1D – continued (full blots with HT-29 lysate as a positive control)

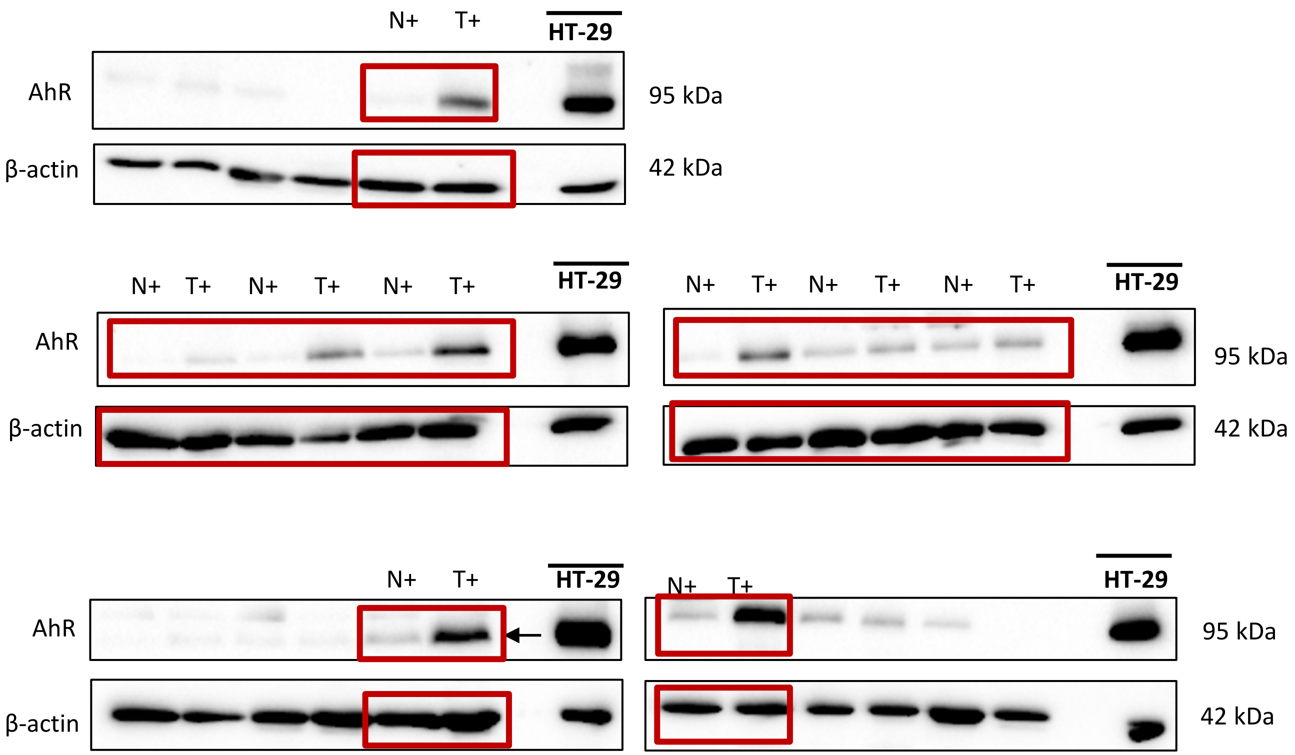

Figure 2A

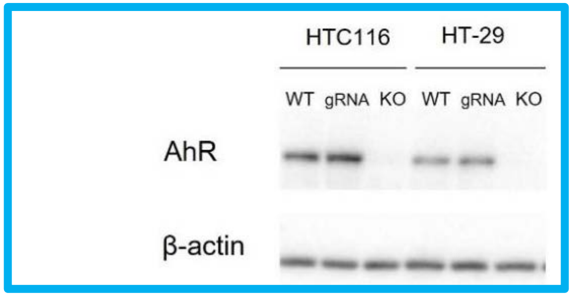

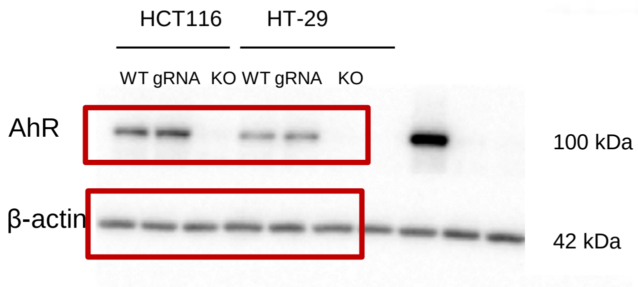

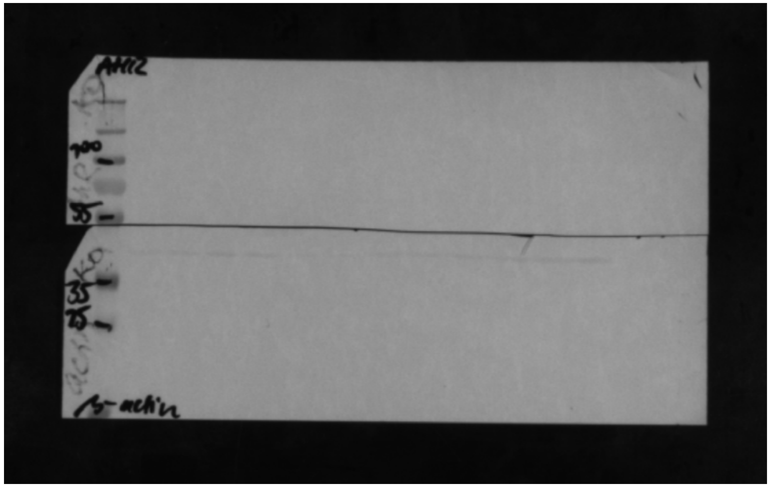

AhR	14.4	20.2	1.6	9.9	11.1	1.3	0.6	39.5	0.8	0.5
β-actin	8.3	8.5	8.6	10.8	10.1	9.2	7.7	10.6	12.5	13.5

% peak area

AhR

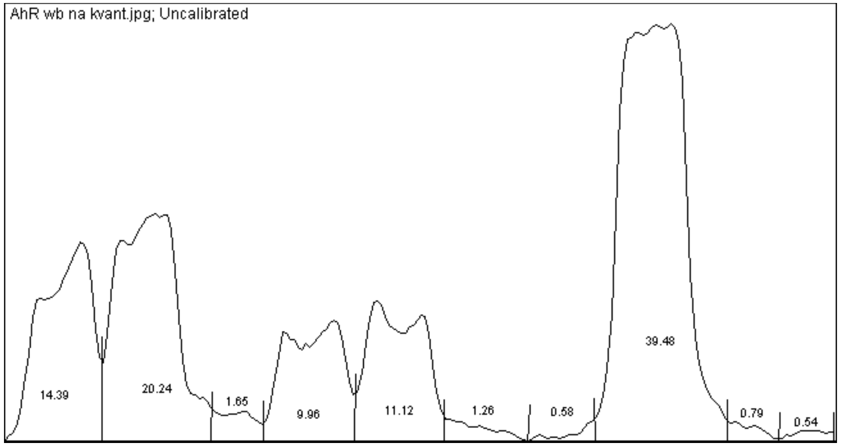

β-actin

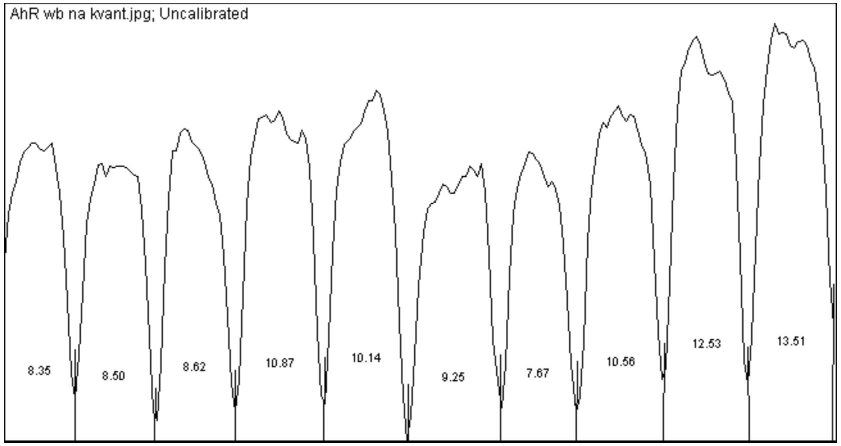

Figure 5D

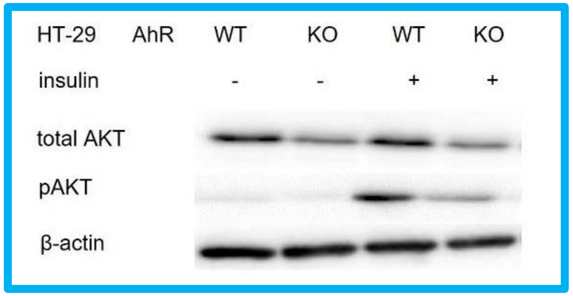

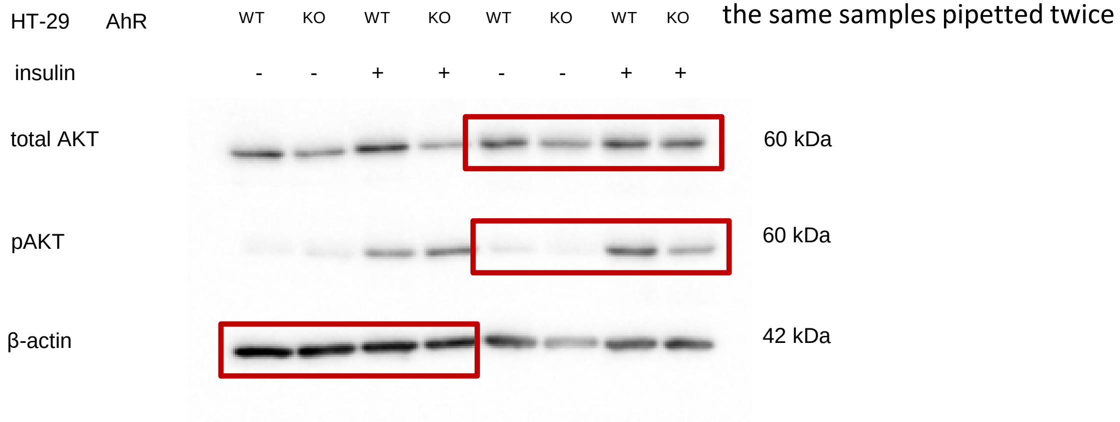

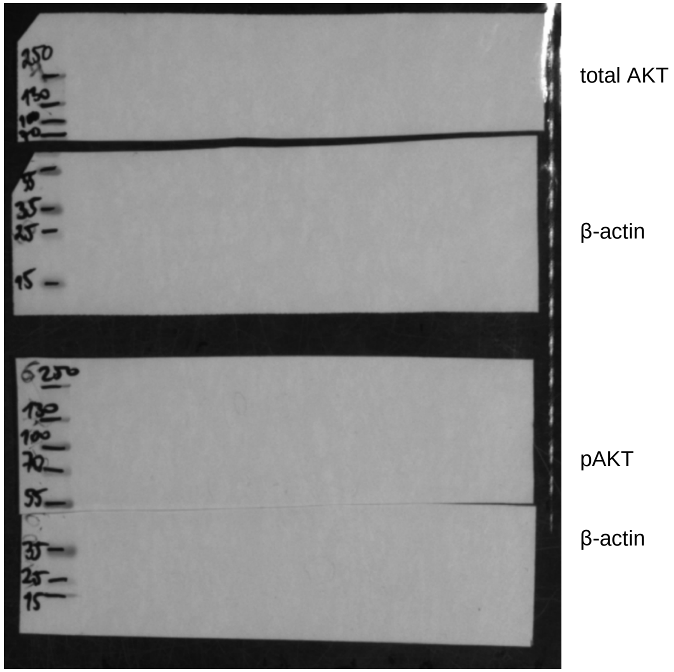

total AKT	30	19,8	36,2	14,1
pAKT	8,5	5,8	56,0	29,7
β-actin	23	22,3	28,7	25,8

% peak area

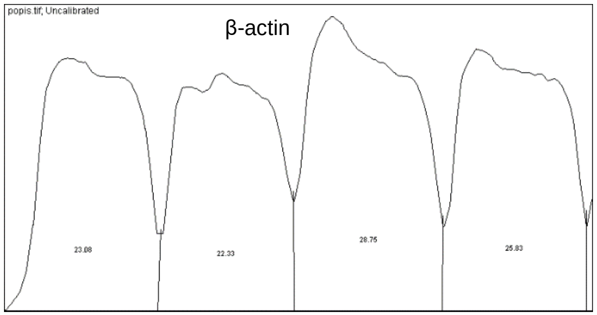

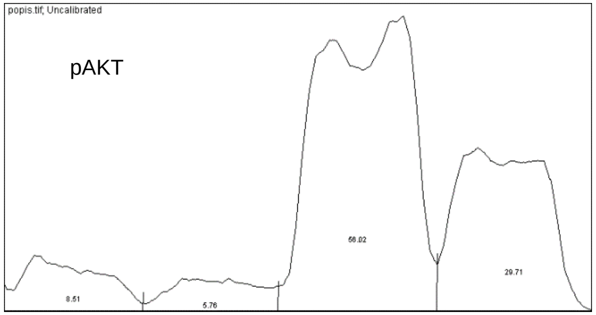

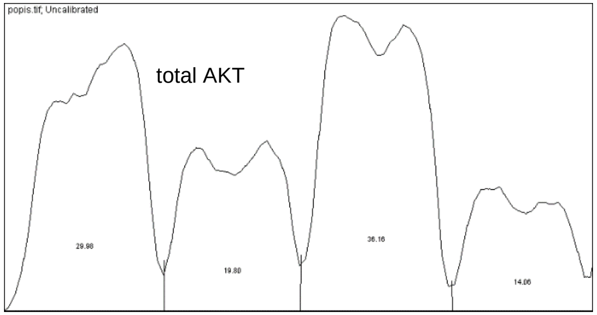

Figure 5D - continued

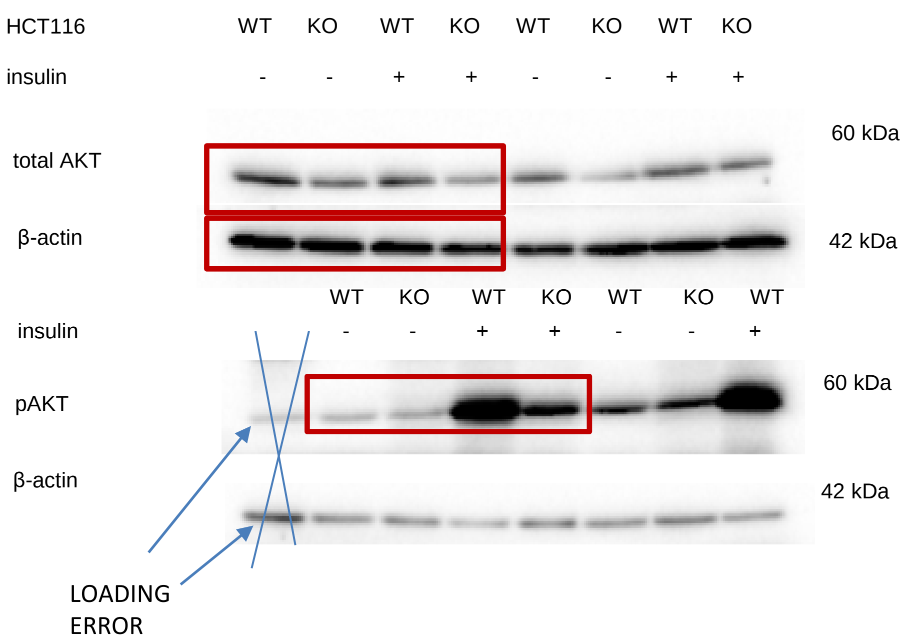

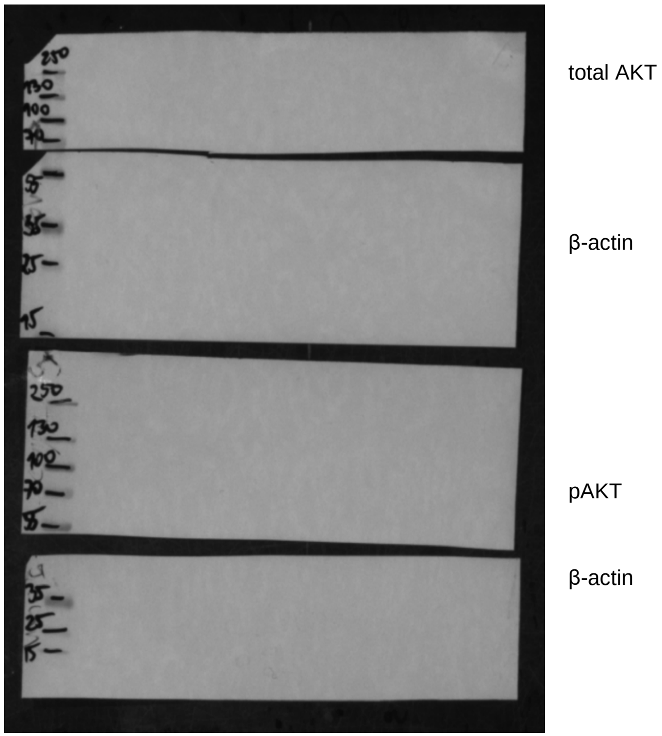

total AKT	39.12	21.18	26.79	12.90
pAKT	3.36	9.85	58.96	27.82
β-actin	27.88	27.40	23.99	20.73

% peak area

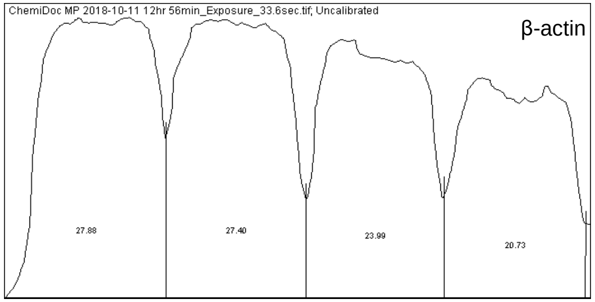

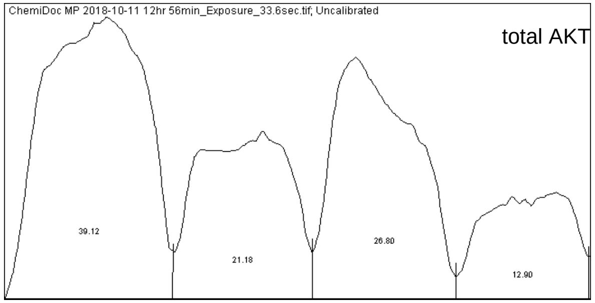

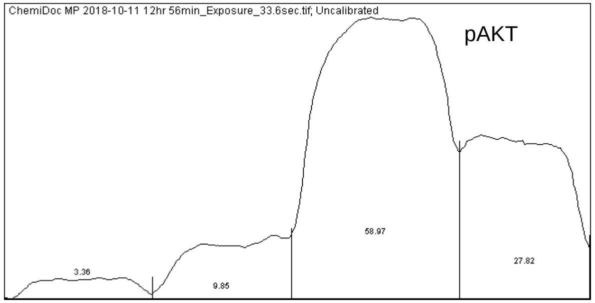

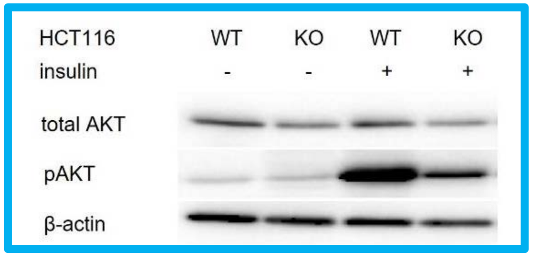

Figure 6B

HCT116	WT	WT	KO	WT	WT	KO	WT	WT	KO	WT	WT	KO
CH-223191	-	+	-	-	+	-	-	+	-	-	+	-
insulin	-	-	-	+	+	+	-	-	-	+	+	+

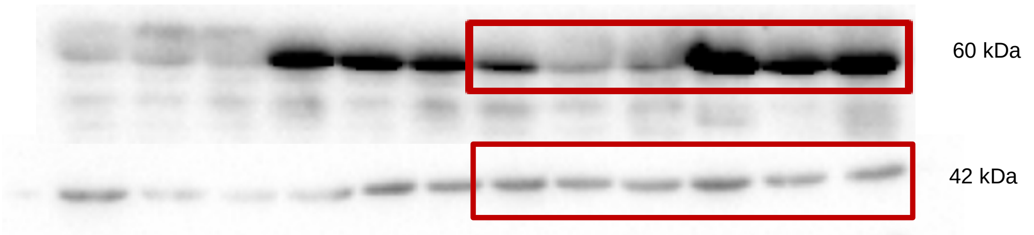

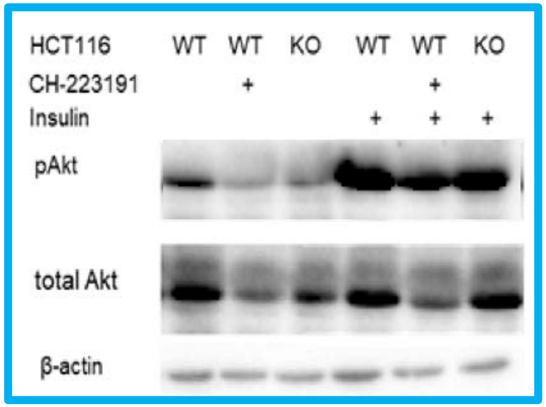

	WT	WT	KO	WT	WT	KO	WT	WT	KO
CH-223191	-	+	-	-	+	-	-	+	-
insulin	-	-	-	+	+	+	+	+	+

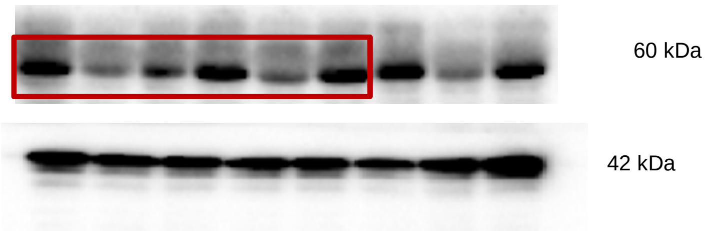

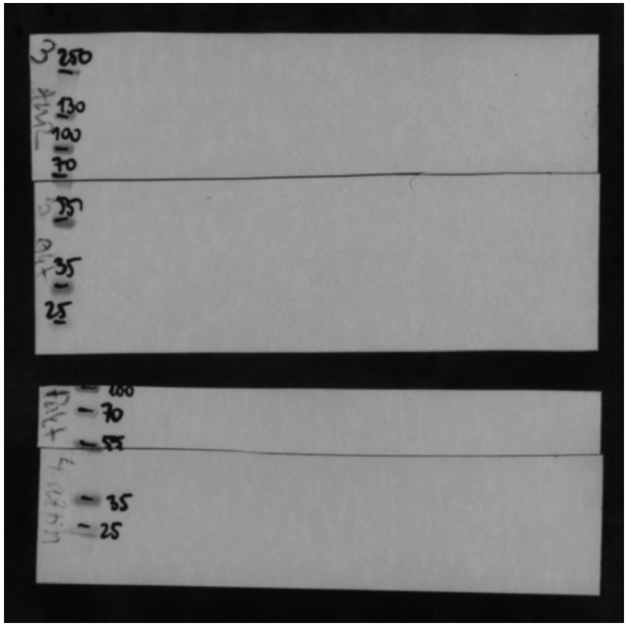

pAKT	9.90	4.34	9.22	31.26	19.92	25.33
total AKT	18.87	14.56	20.5	17.38	11.42	17.70
β-actin	19.50	14.49	14.11	22.47	13.20	16.19

% peak area

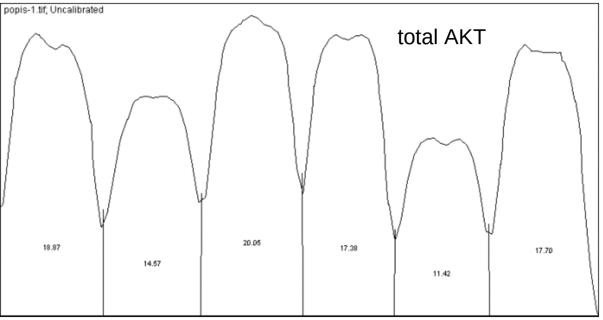

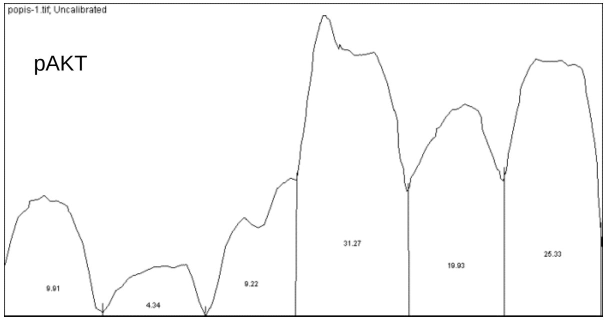

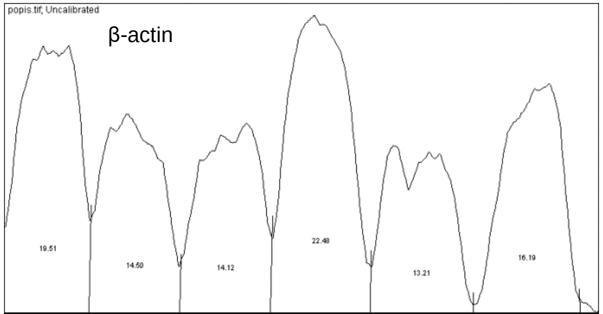

Supplementary Figure S2

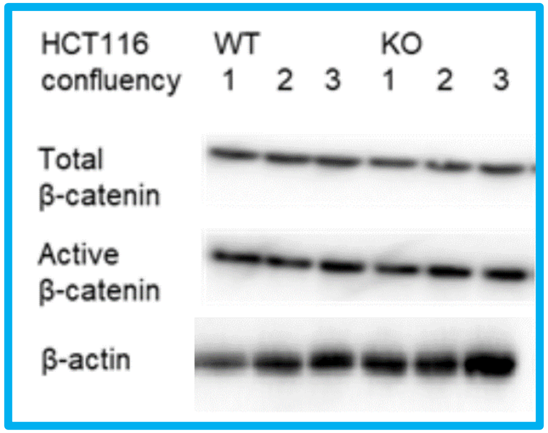

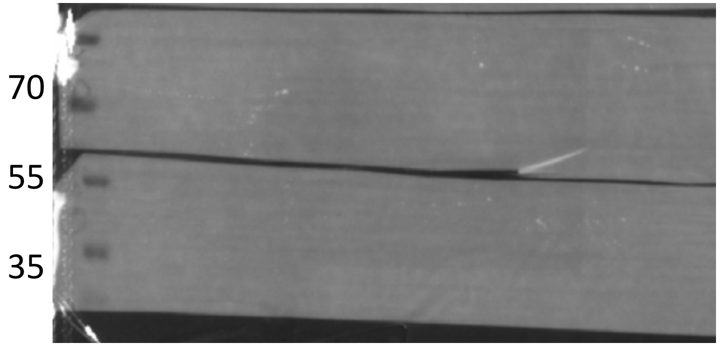

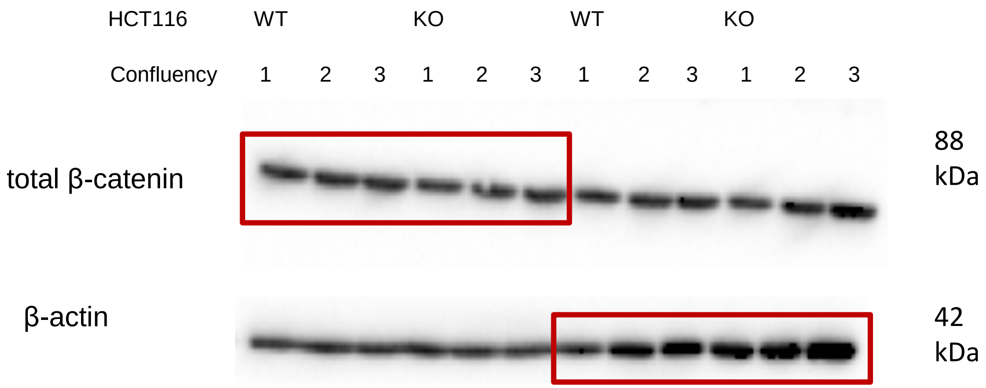

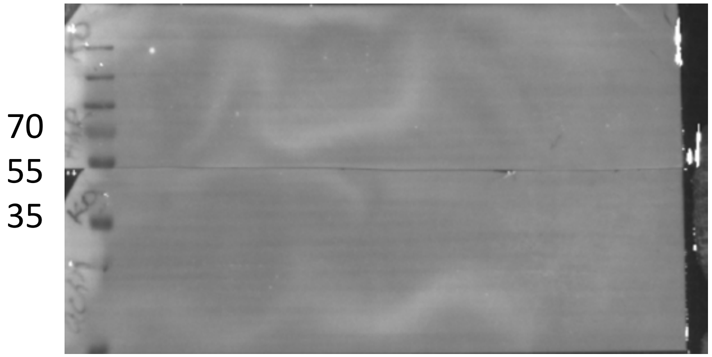

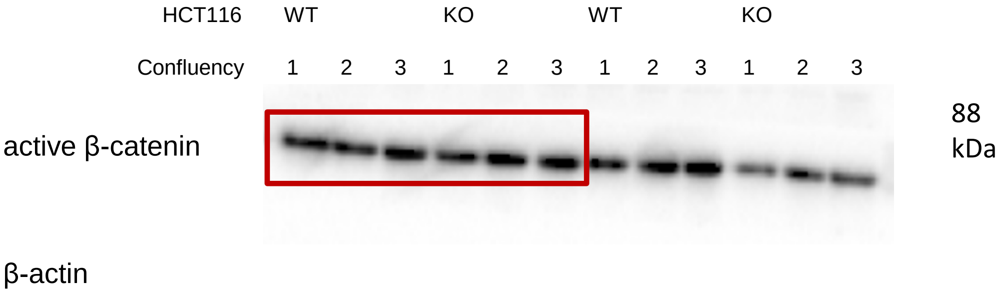

Detection of β-catenin, active β-catenin and β-actin was performed on independently analyzed membranes.
